# On-target, dual aminopeptidase inhibition provides cross-species antimalarial activity

**DOI:** 10.1128/mbio.00966-24

**Published:** 2024-05-08

**Authors:** Rebecca C. S. Edgar, Tess R. Malcolm, Ghizal Siddiqui, Carlo Giannangelo, Natalie A. Counihan, Matthew Challis, Sandra Duffy, Mrittika Chowdhury, Jutta Marfurt, Madeline Dans, Grennady Wirjanata, Rintis Noviyanti, Kajal Daware, Chathura D. Suraweera, Ric N. Price, Sergio Wittlin, Vicky M. Avery, Nyssa Drinkwater, Susan A. Charman, Darren J. Creek, Tania F. de Koning-Ward, Peter J. Scammells, Sheena McGowan

**Affiliations:** 1 School of Medicine, Deakin University, Geelong, Australia; 2 The Institute for Mental and Physical Health and Clinical Translation, Deakin University, Geelong, Australia; 3 Monash Biomedicine Discovery Institute and Department of Microbiology, Monash University, Clayton, Australia; 4 Drug Delivery, Disposition and Dynamics, Monash Institute of Pharmaceutical Sciences, Monash University, Parkville, Australia; 5 Discovery Biology, Centre for Cellular Phenomics, Griffith University, Nathan, Queensland, Australia; 6 Global Health and Tropical Medicine Division, Menzies School of Health Research, Charles Darwin University, Darwin, Northern Territory, Australia; 7 Eijkman Institute for Molecular Biology, Jakarta, Indonesia; 8 Centre for Tropical Medicine and Global Health, Nuffield Department of Clinical Medicine, University of Oxford, Oxford, United Kingdom; 9 Mahidol-Oxford Tropical Medicine Research Unit, Faculty of Tropical Medicine, Mahidol University, Bangkok, Thailand; 10 Swiss Tropical and Public Health Institute, Allschwil, Switzerland; 11 University of Basel, Basel, Switzerland; 12 School of Environment and Science, Griffith Sciences, Griffith University, Nathan, Queensland, Australia; 13 Centre for Drug Candidate Optimisation, Monash Institute of Pharmaceutical Sciences, Monash University, Parkville, Australia; 14 Medicinal Chemistry, Monash Institute of Pharmaceutical Sciences, Monash University, Parkville, Australia; NIAID/NIH, Rockville, Maryland, USA

**Keywords:** malaria, drug resistance, aminopeptidase, *Plasmodium falciparum*, *Plasmodium vivax*

## Abstract

To combat the global burden of malaria, development of new drugs to replace or complement current therapies is urgently required. Here, we show that the compound **MMV1557817** is a selective, nanomolar inhibitor of both *Plasmodium falciparum* and *Plasmodium vivax* aminopeptidases M1 and M17, leading to inhibition of end-stage hemoglobin digestion in asexual parasites. **MMV1557817** can kill sexual-stage *P. falciparum*, is active against murine malaria, and does not show any shift in activity against a panel of parasites resistant to other antimalarials. **MMV1557817**-resistant *P. falciparum* exhibited a slow growth rate that was quickly outcompeted by wild-type parasites and were sensitized to the current clinical drug, artemisinin. Overall, these results confirm **MMV1557817** as a lead compound for further drug development and highlights the potential of dual inhibition of M1 and M17 as an effective multi-species drug-targeting strategy.

**IMPORTANCE:**

Each year, malaria infects approximately 240 million people and causes over 600,000 deaths, mostly in children under 5 years of age. For the past decade, artemisinin-based combination therapies have been recommended by the World Health Organization as the standard malaria treatment worldwide. Their widespread use has led to the development of artemisinin resistance in the form of delayed parasite clearance, alongside the rise of partner drug resistance. There is an urgent need to develop and deploy new antimalarial agents with novel targets and mechanisms of action. Here, we report a new and potent antimalarial compound, known as **MMV1557817**, and show that it targets multiple stages of the malaria parasite lifecycle, is active in a preliminary mouse malaria model, and has a novel mechanism of action. Excitingly, resistance to **MMV15578117** appears to be self-limiting, suggesting that development of the compound may provide a new class of antimalarial.

## INTRODUCTION

Malaria remains a leading cause of global mortality, with over 600,000 deaths estimated in 2022 due to infection with *Plasmodium* spp., the majority of these caused by *P. falciparum* and/or *P. vivax* (1). For the past decade, artemisinin-based combination therapies (ACTs) have been recommended by the World Health Organization (WHO) as the standard malaria treatment worldwide. Their widespread use has led to the development of artemisinin partial resistance in the form of delayed parasite clearance, most notably within southeast Asia and more recently within highly endemic regions of Africa (2–4). Continued emerging resistance in both artemisinin and partner drug(s) jeopardizes the elimination milestones set by the WHO (1) and highlights the urgent need to develop and deploy new antimalarial agents with novel targets and mechanisms of action.

The *Plasmodium* metallo-aminopeptidases M1 and M17 are potential targets worthy of antimalarial development (5–11). These exo-peptidases function at the terminal stages of intra-erythrocytic hemoglobin digestion, a process that occurs in a specialized digestive vacuole (DV) within the parasite and which is required for survival (12). These enzymes utilize a metal-dependent mechanism to facilitate hydrolysis of the N-terminal residue from small peptide substrates (13, 14). The M1 aminopeptidase enzymes have a broad substrate specificity and can cleave N-terminal basic or hydrophobic residues from a peptide substrate (15, 16). In contrast, the *Plasmodium* M17 aminopeptidase displays a restricted specificity and generally only acts on peptides with an N-terminal leucine or tryptophan residue. Although M1 and M17 enzymes have distinct structural and functional features, their overall mechanism of action is the same, with both possessing their own S1 and S1’ substrate-binding pockets and essential divalent zinc ion(s) in their catalytic site (17, 18).

Both M1 and M17 appear to be essential for survival in *P. falciparum* parasites, making them attractive potential drug targets. Selective inhibition of *Pf*A-M1 with a specific activity-based probe resulted in swelling of the DV and parasite death (5, 8), and attempts to knockout the gene have not been successful (19, 20). While it was previously reported that inhibition of *Pf*A-M17 with a specific activity-based probe resulted in ring-stage parasite death, this appears to be due to off-target effects, with genetic knockdown of *Pf*A-M17 resulting in parasite death at the later trophozoite stage (7). Importantly, inhibition of *Pf*A-M1 or knockdown of *Pf*A-M17 expression leads to a build-up of undigested short peptide chains that likely originate from hemoglobin, providing confirmation of their function (7, 8). Multiple studies performed in the murine model *Plasmodium chabaudi chabaudi* have also confirmed that inhibition of these aminopeptidases reduces parasite burden, confirming their druggability in an *in vivo* model (5, 21, 22).

Since inhibition of both M1 and M17 leads to parasite death, we developed potent inhibitors that target both enzymes (6, 9, 11). We have previously reported the synthesis and initial characterization of **MMV1557817** (*N*-(2-(hydroxyamino)−2-oxo-1-(3′,4′,5′-trifluoro-[1,1′-biphenyl]-4-yl)ethyl)-3,3-dimethylbutanamide). This compound was found to dually inhibit M1 and M17 aminopeptidases from both *P. falciparum* and *P. vivax* in recombinant assays and exhibited antiplasmodial activity against *P. falciparum* (11). In this current study, we report the pre-clinical characterization of **MMV1557817** and investigate its mechanism of action. **MMV1557817** was effective against *P. falciparum* parasites that are resistant to a wide range of other antimalaria drugs as well as sexual-state parasites. **MMV1557817** was also effective against *P. falciparum* and *P. vivax* clinical isolates cultured *ex vivo* and showed promising exposure *in vivo* and could effectively clear a *Plasmodium berghei* infection. We confirmed that **MMV1557817** is on target for both *Pf*A-M1 and *Pf*A-M17 and found that parasites made resistant to **MMV1557817** had a significantly slower growth rate and an increase in hemoglobin digestion. Overall, these results confirm that **MMV1557817** is a candidate for further development as a novel and promising lead compound, while also validating M1 and M17 aminopeptidases as suitable cross-species targets for novel drug development.

## MATERIALS AND METHODS

### Recombinant enzyme assays

Inhibition of aminopeptidase activity assays were performed against nine aminopeptidases: the M1 alanyl and M17 leucyl aminopeptidases from *P. falciparum* (*Pf*A-M1; *Pf*A-M17), *P. vivax* (*Pv-*M1; *Pv-*M17), and *P. berghei* (*Pb*-M1; *Pb*-M17), and three human M1 homologs: LTA4H (OriGene TP307617), ERAP1 (OriGene TP314469), and ERAP2 (Creative BioMart ERAP2-304H). The *Plasmodium* enzymes were produced recombinantly as described previously (16, 17), and the human recombinant enzymes were purchased from commercial suppliers as indicated.

The ability of **MMV1557817** to inhibit aminopeptidase activity was assessed by fluorescence assays using fluorogenic substrates *L*-Leucine-7-amido-4-methylcoumarin hydrochloride (H-Leu-NHMec) for all enzymes except LTA4H, which was assessed using *L*-alanine-7-amido-4-methylcoumarin hydrochloride (H-Ala-NHMec, 20 µM). The concentration of H-Leu-NHMec in the assay was held constant for each enzyme and ranged from 10 to 100 µM, depending on the enzyme. Reactions were measured at 37°C in white 384-well plates in a total volume of 25 or 50 µL using a spectrofluorimeter (BMG FLUOstar) with excitation at 355 nm and emission at 460 nm. The fluorescence signal was continuously monitored until a final steady-state velocity, *v*, was obtained. Inhibition constants were calculated from biological triplicates made from three different protein preparations.

For determination of the Morrison inhibition constant (*K*_i_^app^), enzymes were pre-incubated in 100 mM Tris-HCl, pH 8.0 (supplemented with 1 mM CoCl_2_ for M17 aminopeptidases), and the inhibitors added 20 min prior to the addition of substrate. Substrate concentrations were selected to allow sensitive detection of enzyme activity while not exceeding the *K*_m_ for each enzyme. A compound concentration range was selected to obtain a complete inhibition curve (0%–100 %) in biological triplicate. Where possible, the *K*_i_ values were calculated by plotting the initial rates versus inhibitor concentration and fitting to the Morrison equation for tight-binding inhibitors in GraphPad Prism (V.8.4.1; non-linear regression method). Where a *K*_i_^app^ could not be calculated, the percentage inhibition was calculated assuming 100% activity in the absence of compound.

### Safety pharmacology screening

**MMV1557817** was screened at 10 µM for % inhibition against known human DNA repair (including HDAC1, 2, 5, 7, 8, 9, and 10 and sirtuins 3 and 6) and matrix metalloprotease (MMP2, 3, 7, 8, 9, and 13 and MT1) enzymes by Eurofins Cerep (study number 100048667 and 100048668). Results showing any inhibition or stimulation higher than 50% are considered to represent significant effects of the test compounds.

### Protein crystallography

*Pf*A-M1 and *Pf*A-M17 were co-crystallized with **MMV1557817** or crystallized unbound by the hanging-drop method, using previously established protocols (17, 18). Purified protein was concentrated to 5.0 mg/mL and 15 mg/mL respectively and, for co-crystals, mixed with **MMV1557817** to a final ligand concentration of 1 mM. For *Pf*A-M1, crystals grew in 20%–30% poly(ethylene glycol) (PEG)8000, 0.1 M Tris pH 7.5–8.5, 0.2 M MgCl_2_ and 10% glycerol. For *Pf*A-M17, crystals grew in 30%–40% PEG(400), 0.1 M Tris pH 7.5–8.5, and 0.2 M Li_2_SO_4_. Co-crystals were subjected to an additional overnight compound soak (mother liquor supplemented with 1 mM ligand and 1 mM Zn^2+^ for *Pf*A-M17) before being snap frozen in liquid nitrogen. Data were collected at 100 K using synchrotron radiation at the Australian Synchrotron beamlines 3BM1 (23) and 3ID1. Data was processed using iMosflm (24) or XDS (25) and Aimless (26) as part of the CCP4i program suite (27). The structures were solved by molecular replacement in Phaser (28) using the structure of unliganded enzymes (RCSB ID 3EBG for *Pf*A-M1 and 3KQZ for *Pf*A-M17) as the search models. The structures were refined using Phenix (29), with 5% of reflections set aside for calculation of *R*_free_. Between refinement cycles, the protein structure, solvent, and inhibitors were manually built into 2*F*_o_ − *F*_c_ and *F*_o_ − *F*_c_ electron density maps using COOT (30, 31), with restraint files generated by Phenix where necessary. The data collection and refinement statistics can be found in Table S1. The coordinates and structure factors are available from the Protein Data Bank with PDB accession codes: 8SVL (*Pf*A-M1-**MMV1557817**), 8SVM (*Pf*A-M17-**MMV1557817**), and 8SW9 [*Pf*A-M17(A460S)].

### Parasite cultures

*P. falciparum* asexual blood-stage parasites were cultured in human O^+^ red blood cells (RBCs) at 3%–5% hematocrit in RPMI-1640 media supplemented with 25 mM HEPES, 50 µM hypoxanthine, 2 mM L-glutamine, 2 g/L sodium bicarbonate, 0.5% (wt/vol) AlbuMAXII (Invitrogen), and 10 µg/mL gentamicin, in modular incubator chambers at 5% O_2_ and 1% CO_2_ in N_2_ at 37°C unless otherwise stated. Human RBCs and serum used for *P. falciparum in vitro* culturing and serum used for *ex vivo* culturing of *P. falciparum* and *P. vivax* were obtained from the Australian Red Cross Blood Service.

### Determination of MMV1557817 EC_50_ in laboratory conditions against asexual-stage parasites

Viability assays were performed as previously described (7). Sorbitol-synchronized *Pf*3D7 (a laboratory strain which is broadly sensitive to standard antimalarial drugs) at ring stages was cultured in the presence of drug serially diluted across a 96-well plate at 0.5% parasitemia and 2% hematocrit. Cultures were grown for 72 h before being placed at −80°C. After thawing, cultures were incubated with equal volumes of lysis buffer (20 mM Tris [pH 7.5], 5 mM EDTA, 0.008% [wt/vol] saponin, and 0.008% [vol/vol] Triton x-100) containing 0.2 µL/mL SYBR Green I Nucleic Acid Gel Stain (10,000× in dimethyl sulfoxide [DMSO]; Thermo Fisher). After 1 h incubation, fluorescence intensity was read on a Glomax Explorer Fully Loaded (Promega) at emission wavelengths of 500–550 nm and an excitation wavelength of 475 nm. Graphs were generated using GraphPad Prism (V.8.4.1). Parasite survival was compared with vehicle-treated cultures in three biological replicates performed in triplicate. This EC_50_ was used in asexual *P. falciparum* assays unless otherwise stated.

### Determination of MMV1557817 IC_50_ against sexual-stage parasites

Viability assays were performed as described previously (32, 33) against *Pf*NF54-*s16*-GFP early(I–III), late (IV–V), and mature (V) stage gametocytes. Gametocytes were assessed at the appropriate time points following gametogenesis; early stage (day 2), late stage (day 8), and mature stage (day 12). Compounds diluted in 4% DMSO were transferred into 384-well imaging plates; gametocytes prepared as described previously (35) were added, and plates were incubated for 72 h in 5% CO_2_, 5% O_2_, and 60% humidity at 37°C. After 72 h incubation, 5 µL of MitoTracker Red CMH2XRos in phosphate buffered saline (PBS) was added per well, and plates were incubated overnight. Image acquisition and analysis were undertaken on the Opera QEHS micro-plate confocal imaging system. An Acapella-based script using the CMH2XRos fluorescent signal and the GFP-designated object quantifying viable stage-dependent parasite morphology identified gametocytes. Gametocyte viability was calculated as a percentage of the positive (5 µM puromycin) and negative (0.4% DMSO) controls. IC_50_ values were calculated using a 4-parameter log dose, non-linear regression analysis, with sigmoidal dose response (variable slope) curve fit using GraphPad Prism (ver 4.0). No constraints were used in the curve fit. Chloroquine, artesunate, pyronaridine, pyrimethamine, dihydroartemisinin (DHA), and methylene blue were used as control compounds. Experiments were performed as two or three biological replicates in duplicate.

### MMV1557817 EC_50_ against drug-resistant field isolates and laboratory-selected *P. falciparum* parasites

*In vitro* testing was performed using the modified [^3^H]hypoxanthine incorporation assay, as previously reported (34). The specific mutations in the field isolates (see Table 1, top six strains) have been previously described (35, 36), as have the specific mutations in the laboratory-selected Dd2 parasites (see Table 1, remaining five strains) (36).

### Parasite killing rate assay

Assays were performed as previously described (7). *Pf*3D7 parasite cultures at the ring or trophozoite stage were set up at 0.5% parasitemia and 2% hematocrit in a 96-well plate and treated with 10× EC_50_ of **MMV1557817** or artesunate for 24 or 48 h. Parasite cultures incubated for 48 h were fed at 24 h with fresh media containing compound. The compound was then washed out with 3× washes, and cultures were serially diluted 1/3 before being allowed to grow for a further 48 h, after which time the plates were placed at −80°C. Once thawed, cultures were analyzed using SYBR Green I as described above. Parasite viability was determined as a percentage of vehicle-treated controls, and experiments were performed in four biological replicates.

### Parasite reduction ratio

Assays were performed as described previously with some alterations (37). *Pf*3D7 parasite cultures at 0.5% parasitemia and 2% hematocrit were treated with 10× the EC_50_ of **MMV1557817** or chloroquine in 96-well plates. Specifically, media containing compound was placed in wells every 24 h for up 120 h. After each of the 5 treatment days (0 h, 24 h, 48 h, 72 h, 96 h, or 120 h), a subset of wells was removed and the drug was washed off with 3× washes, after which the parasites were serially diluted 1/3. The cultures were then maintained for a further 3 weeks, during which time the parasites were fed with complete culture medium three times a week. Parasite growth was measured at day 21 using SYBR green I as described above. Fluorescence was used to determine at what treatment day wells became positive for growth when compared with the well before it; greater than double the fluorescence was determined to be a positive well. This data was then transformed into a log (parasite viability) + 1 value for each day and plotted on GraphPad Prism (V.8.4.1). Experiments were performed in two or three biological replicates in quadruplicate.

### *Ex vivo* activity of MMV1557817 against drug-resistant field isolates

Ethical approval for this study was obtained from the Eijkman Institute Research Ethics Commission, Eijkman Institute for Molecular Biology, Jakarta, Indonesia (EIREC-47), and the Human Research Ethics Committee of the Northern Territory (NT) Department of Health & Families and Menzies School of Health Research, Darwin, Australia (HREC 2010-1396).

*Plasmodium* isolates were collected from patients attending public health clinics in Timika (Papua, Indonesia), a region endemic for multidrug-resistant strains of *P. vivax* and *P. falciparum* (38–40). Patients with symptomatic malaria presenting to an outpatient facility were recruited into the study if infected with either *P. falciparum* or *P. vivax*, with a parasitemia of between 2,000 µL^−1^ and 80,000 µL^−1^, and the majority (>60%) of asexual parasites at the ring stage of development. Venous blood (5 mL) was collected by venipuncture, and after removal of patient white blood cells using Plasmodipur filters (EuroProxima B.V., The Netherlands), packed infected red blood cells (iRBCs) were used for the *ex vivo* drug susceptibility assay.

Anti-malarial drugs chloroquine (CQ), piperaquine (PIP), mefloquine (MFQ), and artesunate (AS) (WWARN QA/QC Reference Material Programme) and **MMV1557817** were prepared as 1 mg/mL stock solutions in H_2_O or DMSO. Drug plates were pre-dosed by diluting the compounds in 50% methanol followed by lyophilization and storage at 4°C. Drug susceptibility of *P. vivax* and *P. falciparum* isolates was measured using a protocol modified from the WHO microtest as described previously (40, 41). Briefly, 200 µL of a 2% hematocrit blood media mixture (BMM), consisting of RPMI 1640 medium plus 10% AB^+^ human serum (*P. falciparum*) or McCoy’s 5A medium plus 20% AB^+^ human serum (*P. vivax*) was added to each well of pre-dosed drug plates containing 11 serial concentrations (2-fold dilutions) of the anti-malarials; maximum concentrations are shown in brackets: CQ (2,993 nM), PIP (1,029 nM), MFQ (338 nM), AS (49 nM), and **MMV1557817** (356 nM). A candle jar was used to mature the parasites at 37.0°C for 35–56 h. Incubation was stopped when >40% of ring-stage parasites had reached the mature schizont stage in the drug-free control wells as determined by light microscopy. Thick blood films made from each well were stained with 5% Giemsa solution for 30 min and examined microscopically. The number of schizonts per 200 asexual-stage parasites was determined for each drug concentration and normalized to the control well without drug. The dose response data were analyzed using non-linear regression analysis, and the EC_50_ value was derived using an inhibitory sigmoid E_max_ model (*In Vitro* Analysis and Reporting Tool [IVART] [42, 43]). *Ex vivo* EC_50_ data were only used from predicted curves where the *E*_max_ and *E*_0_ were within 15% of 100 and 1, respectively.

### Determination of MMV1557817 and artemisinin interaction *in vitro*

Fractional inhibitory concentrations (FIC) were determined as previously described (44). The EC_50_ values were calculated from fixed mixed ratios (5:0 to 0:5) of artemisinin (starting dilution 100 nM) and **MMV1557817** (starting dilution 1,000 nM) as described above. The FIC for each drug was determined by EC_50_ of drug in a mixture/EC_50_ of drug alone, and the sum of FIC was determined by the addition of the two individual FIC values. Synergistic FIC is described as <1, additive = 1 < 2, and antagonism > 2 (44).

### *In vitro* ADME and *in vivo* exposure in mice

Preliminary studies were conducted to assess the *in vitro* absorption, distribution, metabolism, and excretion (ADME) properties and *in vivo* mouse exposure of **MMV1557817** following single oral dosing. Kinetic solubility was conducted using nephelometry based on a modification of a previously published method (45). Briefly, compound was dissolved in DMSO and spiked into either pH 6.5 phosphate buffer (to reflect the pH of the fasted state upper small intestine) or 0.01 M HCl (to reflect gastric pH 1.5–2) with a final DMSO concentration of 1% (vol/vol) and final concentrations ranging from 1 to 100 µg/mL. Samples were analyzed by nephelometry to determine the concentration above which precipitation occurred.

Metabolic stability was assessed by incubating **MMV1557817** (1 µM) with human, rat, or mouse liver microsomes (0.4 mg/mL) over 60 min at 37°C in the absence or presence of an NADPH-regenerating buffer. Samples were collected over the incubation period, quenched by the addition of an equal volume of acetonitrile, and analyzed using a Waters Acquity UPLC and Xevo G2 QTOF mass spectrometer with positive electrospray ionization under the MS^E^ mode. The natural log of the substrate concentration was plotted against the incubation time to determine the first-order degradation rate constant which was normalized to the microsomal protein concentration to give the *in vitro* intrinsic clearance (*in vitro* CL_int_, µL/min/mg microsomal protein). Additional metabolism (1 µM substrate) and metabolite identification (10 µM substrate) studies were conducted by incubating **MMV1557817** with human or rat cryopreserved hepatocytes (1.4 × 10^6^ viable cells/mL) suspended in pH 7.4 Krebs-Henseleit buffer over 120 min at 37°C in 7.5% CO_2_. Cell viability was determined by Trypan Blue exclusion. At various times over the incubation period, samples were quenched with the addition of an equal volume of acetonitrile and analyzed as described above. Metabolite identification was conducted by accurate mass and tandem mass spectrometry (MS/MS) analysis.

The stability of **MMV1557817** was further assessed by incubating with mouse (purchased from the Animal Resources Centre, Perth, Western Australia) or human (obtained from the Australian Red Cross Blood Service) plasma over 4 h at 37°C. Plasma was spiked with **MMV1557817** (~500 ng/mL), and samples were taken periodically, and proteins were precipitated with a twofold excess of acetonitrile. After centrifugation, the supernatant was collected and stored at −80°C until analysis. Sample analysis was conducted using a Waters Acquity UPLC and Quattro Premier mass spectrometer operated in a positive electrospray ionization mode with multiple-reaction monitoring (transition [*m*/*z*] 395.27 > 362.28, cone voltage 20 V, CID 7 V). The column was a Supelco Ascentis Express RP Amide (50 × 2.1 mmm 2.7 µm), and the mobile phase was a water/acetonitrile gradient containing 0.05% formic acid with a 4 min cycle time. The injection volume was 3 µL, and the flow rate was 0.4 mL/min. Concentrations were determined by comparison to a calibration curve prepared in blank plasma and processed in the same way as for the samples.

Plasma protein binding (human, rat, and mouse plasma) and binding to the Albumax medium used for *in vitro* activity assessment were measured by ultracentrifugation based on a method reported previously (46) under conditions that maintained the pH at 7.4 ± 0.1. Human plasma was obtained from the Volunteer Blood Donor Registry (Walter & Eliza Hall Institute, Melbourne, Australia), rat plasma was from male Sprague-Dawley rats (Animal Resource Centre, Perth, Western Australia) and mouse plasma was procured as described above. RPMI-1640 media supplemented with 25 mM HEPES, 2 g/L sodium bicarbonate, and 100 mg/L neomycin were additionally supplemented with 5 g/L AlbuMAXII on the day prior to binding assessment. Plasma protein binding was measured using 10% plasma diluted with pH 7.4 PBS, and the extent of binding in neat plasma was then calculated via an established approach which accounts for the shift in equilibria that occurs with protein dilution (47). Each medium was spiked with **MMV1557817** (~1,000 ng/mL for plasma, 1 µM for Albumax), briefly mixed, and equilibrated for ~30 min (Albumax equilibration was conducted in a 5% CO_2_ incubator to maintain pH 7.4) after which they were subjected to ultracentrifugation at 37°C using a sealed rotor (Beckman Rotor type 42.2 Ti; 223,000 *g*) for 4.2 h to separate proteins. Additional aliquots of spiked matrix were maintained at 37°C for 4.2 h but not centrifuged to serve as controls for stability assessment and to obtain a measure of the total concentration in each matrix. Following centrifugation, aliquots of the protein-free supernatant and the non-centrifuged matrix controls were “matrix matched” by addition of an equal volume of the opposite medium (i.e., either blank buffer or blank matrix) and assayed as described above with comparison to calibration standards prepared in 50% matrix/50% PBS.

*In vivo* exposure of **MMV1557817** was assessed in non-infected male Swiss outbred mice in parallel to *in vivo* efficacy studies. All animal studies were conducted using established procedures in accordance with the Australian Code of Practice for the Care and Use of Animals for Scientific Purposes, and the study protocols were reviewed and approved by the Monash Institute of Pharmaceutical Sciences Animal Ethics Committee. **MMV1557817** was dissolved in 70% (vol/vol) Tween 80/30% (vol/vol) ethanol and diluted 10-fold with water just prior to dosing. This produced a uniform off-white milky suspension of pH 6.0. **MMV1557817** (50 mg/kg) was dosed by oral gavage (10 mL/kg) to non-fasted mice (29–35 g), and blood samples were collected into heparinized tubes for up to 24 h post-dosing (*n* = 2 mice per time point) with a maximum of two samples per mouse. Samples were collected by either submandibular bleed (~120 µL) or terminal cardiac puncture (under inhaled isoflurane anesthesia). Blood samples were centrifuged immediately, and supernatant plasma was removed and stored at −80°C until analysis as described above.

### Efficacy testing of MMV1557817 *in vivo*

Stock solutions of compounds (**MMV1557817** and artesunate)) were dissolved in 70% (vol/vol) Tween 80/30% (vol/vol) ethanol and diluted 10-fold with water just prior to dosing. Preliminary biological assessment of the *in vivo* antimalaria efficacy of **MMV1557817** was assessed using the *P. berghei* rodent malaria 4-day Peters’ suppressive test (48). Female Balb/c mice at 6 weeks of age in groups of 4–5 were infected intraperitoneally with 2 × 10^7^ *P. berghei* ANKA*-*infected erythrocytes. At 2 h and days 1, 2, and 3 post-infection, mice were orally gavaged with either 50 mg/kg **MMV1557817**, 30 mg/kg artesunate (Sigma), or vehicle control. Parasitemia was assessed by visualizing Giemsa-stained thin blood smears by microscopy and a minimum of 1,000 RBCs was counted. To calculate percentage antimalarial activity, the following formula was used: 100- (mean parasitemia treated/mean parasitemia vehicle control) × 100. These studies were conducted using established procedures in accordance with the Australian Code of Practice for the Care and Use of Animals for Scientific Purposes, and the study protocols were reviewed and approved by the Deakin University Animal Ethics Committee.

### CRISPR-Cas9 editing of *Pfa-m17*

Introduction of the A460S mutation was attempted using methods previously described (49). The CRISPR guide 5′AATGGTAAAACTATAGAAGT was ligated into the *Bbs*I site of the plasmid pDC2-cam-Cas9-U6-hDHFR (49). Additionally, the last 1,373 base pairs from *Pfa-m17* containing the A460S mutation (gct → tct) was synthesized by Integrated DNA Technologies and ligated into the *Aat*II and *Eco*RI sites of the above plasmid; shield mutations to the guide were incorporated to avoid continuous cutting of the genome by Cas9. An identical plasmid was constructed containing a silent mutation at A460 (gct → gcc). Transfection of *Pf*3D7 was performed as previously described (50), and parasites were selected with 2.5 mM WR99210.

### Thermal proteomics profiling of MMV1557817

Parasite isolation and parasite protein solubilization were performed as previously described (7). For experiment 1, parasite lysate was separated into technical replicates: four for DMSO control (0 nM) and three for 300 nM of **MMV1557817** treatment. For experiment 2, parasite lysate was divided into four technical replicates of DMSO control (0 nM) and 1,200 nM of **MMV1557817** treatment. The samples were then incubated at room temperature for 3 min before being thermally challenged by heating at 60°C for 5 min. The denatured protein fraction was then removed via ultracentrifugation at 100,000 × *g* for 20 min at 4°C (Beckman Coulter Optima XE-90–IVD ultracentrifuge with a 42.2 Ti rotor). The soluble fraction was then processed for proteomics analysis as previously described (7, 51). Liquid chromatography-tandem mass spectrometry (LC-MS/MS) was analyzed using the data-independent acquisition mode as previously described (51). Raw files were processed using in-house-generated *P. falciparum* spectral library using Spectronaut 13.0 as previously described (51). The relative abundance of identified proteins was calculated as fold change of drug-treated conditions relative to the 0 nM control for each experiment (only for proteins with intensities greater than 1 × 10^5^ and with a minimum peptide count of 2). Significant proteins determined by Welch’s *t*-test (*P* value < 0.05) and fold change > 1.2, at multiple concentrations of **MMV1557817**, were considered as stabilized proteins and were plotted using paired volcano plots.

### Metabolomics analysis of MMV1557817, MIPS2673, and 3 compared with DMSO control

*Pf*3D7 cultures at 6% parasitemia and 2% hematocrit were subject to double sorbitol synchronization 14 h apart, followed by further incubation for 28–42 h to achieve the desired trophozoite stage (28 h post-infection). Infected RBCs (2 × 10^8^) were treated with 10× the EC_50_ of **MMV1557817** (320 nM), MIPS2673 (1 µM), or **3** (4.53 µM) for 1 h, after which metabolites were extracted, analyzed, and processed as previously described (7). Principal component analysis and hierarchical clustering algorithms were run also in Metaboanalyst (52). Metabolomics data are presented as relative abundance values from 4 to 7 biological replicates. Differences were determined using Welch’s *t*-test where significant interactions were observed. Significance was determined at *P* values < 0.05. The metabolomics data for **3**, MIPS2673, and drug-free controls were reported previously (7, 53). The metabolomics data are available at the NIH Common Fund’s National Metabolomics Data Repository (NMDR) website, the Metabolomics Workbench, https://www.metabolomicsworkbench.org where it has been assigned Study ID ST003144. The data can be accessed directly via its Project DOI: http://dx.doi.org/10.21228/M8172C.

### Blue native PAGE and western blotting

Cultures of parental Dd2 or **MMV1557817**-resistant parasites were grown to trophozoite stage, and 60 mL of culture with greater than 5% parasitemia was lysed with 0.1% (wt/vol) saponin in PBS and washed 3× to remove hemoglobin. Following centrifugation, the parasite pellet was solubilized with 1% (vol/vol) Triton X-100 and then incubated on ice for 30 min. Insoluble material was pelleted by centrifugation at 14,000 × *g* for 30 min at 4°C. The protein concentration of the supernatant was determined by Bradford assay (Bio-Rad). Next, the supernatants were electrophoresed on NativePAGE Novex 3%–12% Bis-Tris protein gels as per the manufacturer’s instructions (Invitrogen). Briefly, proteins were separated at 150–200 V at 4°C until the dye front ran off the gel. Proteins were transferred to methanol-activated polyvinylidene difluoride (PVDF) membrane using a wet-transfer with a constant current of 300 mAmp for 90 min. Membranes were incubated with 8% acetic acid in water for 20 min at room temperature before being rinsed with MilliQ water and dried overnight. Once dried, the membrane was washed in methanol and western blotting was performed. Blots were blocked in 3% (wt/vol) bovine serum albumin in PBS before being probed with rabbit anti-M17 (1:1,000) as previously described (7). Horseradish peroxidase-conjugated secondary antibodies were used (1:10,000; Thermo Scientific), and protein bands were detected using the Clarity ECl Western blotting substrate (Bio-Rad) and imaged on a Bio-Rad ChemiDoc Imaging System.

### Visualization of aminopeptidase activity in live cells using fluorogenic peptide substrates

Aminopeptidase activity within live parasites was determined using a fluorogenic H-Leu-NHMec peptide (Sigma) as described previously (54). Briefly, sorbitol-synchronized parasites at the trophozoite stage were pelleted, washed 2× with complete RPMI media, and incubated with 10 µM H-Leu-NHMec or N-terminally blocked Z-Arg-ArgMec. After 10 min incubation at 37°C, 10 µL of treated parasites was spotted onto a glass slide, covered with a coverslip, and imaged using a 4′,6-diamidino-2-phenylindole (DAPI) filter set on a Nikon Eclipse Ti2 microscope at 100× magnification under oil immersion. To inhibit *Pf*A-M1 activity, washed parasites were treated with MIPS2673 (3.2 µM; 10× EC_50_) (53) for 20 min prior to substrate addition. The relative fluorescence detected within a parasite cytosol was calculated using ImageJ (NIH, version 1.53c) and was expressed relative to the surface area. Images of at least 20 individual parasites across two biological replicates were taken per treatment under identical conditions. Data were plotted using GraphPad Prism (V.8.4.1), and significance was determined using a one-way analysis of variance (ANOVA). Inhibitors and substrates were diluted in DMSO; the total DMSO did not exceed 0.5% of the final volume.

### Parasite hemoglobin fractionation assay

Hemoglobin was fractionated as previously described with some alterations (55). Double sorbitol-synchronized Dd2 or **MMV1557817**-resistant parasites at mid-late trophozoite stage were harvested with 0.1% (wt/vol) saponin in PBS containing 1× protease inhibitors at 4°C for 10 min before undergoing 3× washes in PBS containing 1× protease inhibitors. Pellets were sonicated in 50 µL of water for 5 min after which 50 µL of 0.2 M HEPES (pH 7.5) was added, and the mixture was centrifuged at 1,500 *g* for 20 min. The supernatant was processed further by the addition of 50 µL 4% (wt/vol) SDS and a further 5 min sonication. The samples were then incubated at 95°C for 5 min before addition of 50 µL of 0.3 M NaCl and 50 µL 25% (vol/vol) pyridine in 0.2 M HEPES (pH 7.5) and vortexing; this sample contained the hemoglobin fraction. The pellet was resuspended in 50 µL water and 50 µL 4% (wt/vol) SDS and sonicated for 5 min before being incubated at 95°C for 5 min. To this, 50 µL of 0.2 HEPES (pH 7.5), 50 µL 0.3 M NaCl, and 50 µL 25% (vol/vol) pyridine was added, and the sample was vortexed and then centrifuged at 1,500 × *g* for 10 min; the resulting supernatant contained the heme fraction. The remaining pellet was solubilized in 50 µL water and 50 µL 0.3 M NaOH by sonication for 5 min and incubation at 95°C for 5 min. Finally, 50 µL of 0.2 M HEPES (pH 7.5), 0.3 M HCl, and 50 µL 25% (vol/vol) pyridine was added, and samples were vortexed; this sample contained the hemozoin fraction. The absorbance of samples was measured at 405 nm on a PerkinElmer Ensight Plate Reader.

## RESULTS

### MMV1557817 is active against aminopeptidases from multiple *Plasmodium* spp.

**Fig 1:**
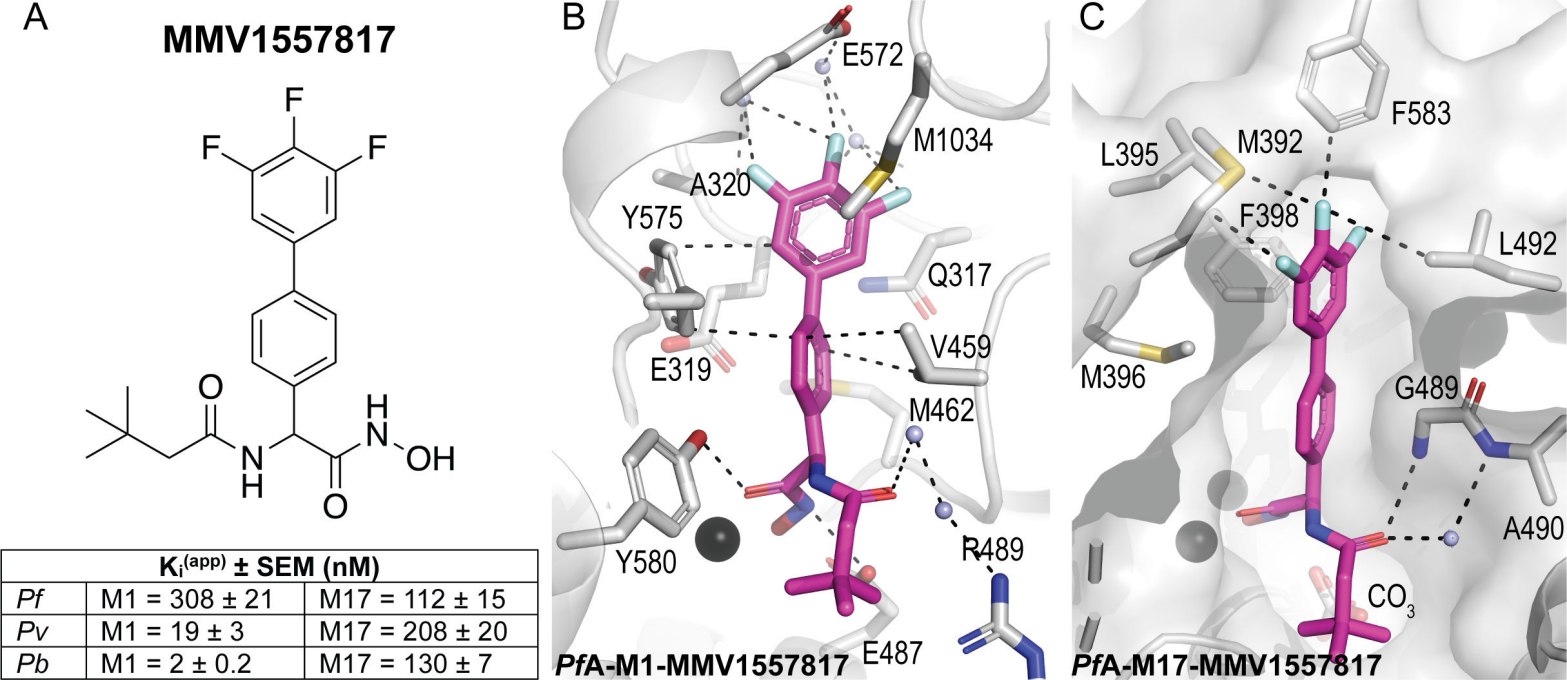
**MMV1557817** is a potent dual inhibitor of *Plasmodium* M1 and M17 aminopeptidases. (**A**) Chemical structure of **MMV1557817**. Inhibition constants (*K*_i_^app^, in nM) for **MMV1557817** toward recombinant, purified *Pf*A-, *Pv*-, and *Pb*-M1 and *Pf*A-, *Pv*-, and *Pb*-M17 are shown underneath panels with the standard error of mean indicated. (**B and C**) Binding mode of **MMV1557817** to *Pf*A-M1 (**B**) and *Pf*A-M17 (**C**). Interactions between **MMV1557817** (magenta sticks) and protein residues (grey) shown as black dashed lines. Residues of interest are indicated.

We confirmed inhibitory constants of **MMV1557817** to be in the nanomolar range for both M1 and M17 aminopeptidases from key *Plasmodium* clinical (*P. falciparum*, *P. vivax*) and animal (*P. berghei*) models (*Pf*A-M1, *Pf*A-M17, *Pv*-M1, *Pv*-M17, *Pb*-M1, and *Pb*-M17; Fig. 1A). The inhibitory constants confirmed earlier studies that had shown *Pf*A-M1 to be more difficult to inhibit than either *Pv* or *Pb* homologs (11, 16). To investigate the binding mode of the compound, data were obtained from co-crystallographic structures of the *P. falciparum* enzymes (*Pf*A-M1, *Pf*A-M17) and was considered representative of the homologous proteins due to the conservation of active site residues and architecture (Table S1) (16). In M1, the 3,4,5-trifluoro biphenyl moiety occupied the S1 subsite with similar interactions as reported previously, including a carbonyl-π with the main chain oxygen of Glu319, edge-face π-stacking with Tyr575, and Met1034 packing against the fluorinated ring (6) (Fig. 1B). The interaction with Met1034 involves movement of the sidechain compared with its unbound position. The S1′ moiety of **MMV1557817** is a 3,3-dimethylbutanamide that occupies a similar position in the S1′ subsite, making limited contacts with residues lining the pocket. For the M17 enzymes, the large 3,4,5-trifluoro biphenyl moiety forms interactions with residues lining both the sides and top of the S1 subsite (Fig. 1C). The methionine side chain situated at the top of the S1 subsite extended away from **MMV1557817**, likely displaced by the bound compound. Variations in this Met sidechain position are likely dependent on the degree of rotation adopted by the biphenyl group. The 3,3-dimethylbutanamide group extends into the S1’ subsite and shows little flexibility.

### MMV1557817 demonstrates potent activity against asexual and sexual stage parasites

**Fig 2:**
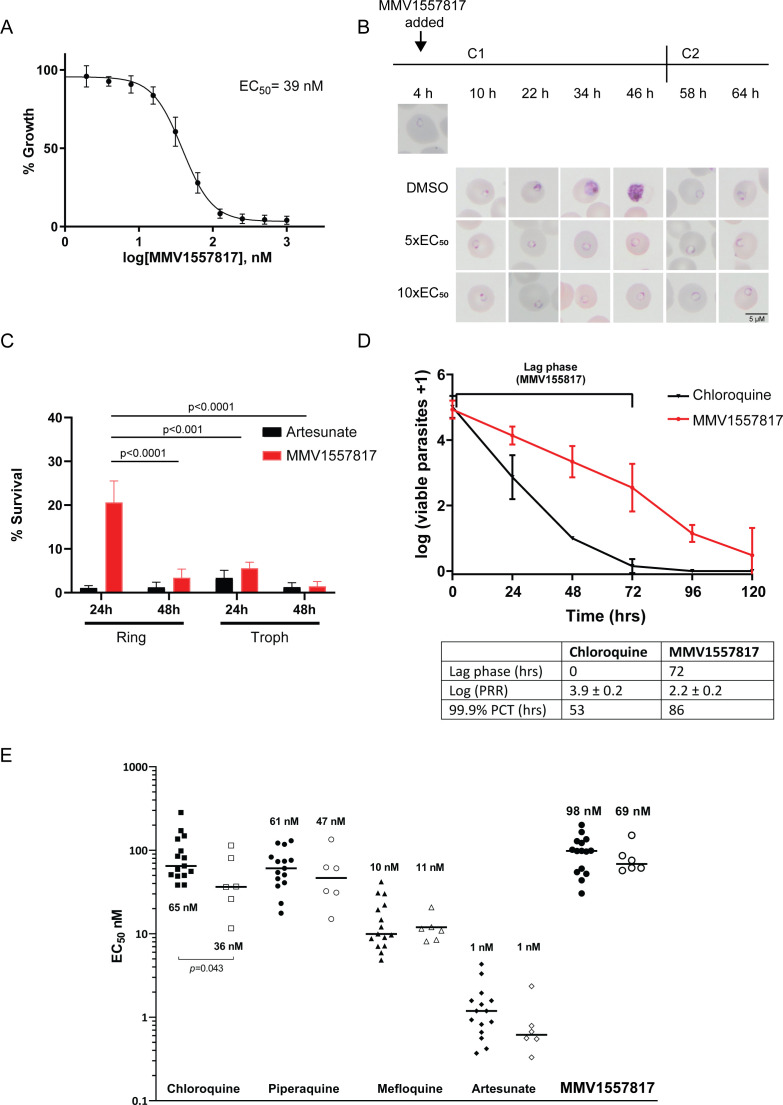
Activity of **MMV1557817** against *P. falciparum* and *ex vivo P. vivax*. (**A**) Killing action of **MMV1557817** determined by a standard 72 h ring killing assay. Plotted is the mean ± standard error of the mean (*n* = 3 performed in triplicate). (**B**) Representative Giemsa-stained *Pf*3D7 parasites treated with **MMV1557817** over two cycles (C1, cycle 1; C2, cycle 2) beginning at 4 h post-infection with 5× or 10× the EC_50_ or DMSO at the concentration present in the 10× EC_50_ treatment. Treated parasites did not progress past ring stage. (**C**) Assessment of **MMV1557817** activity on ring- and trophozoite-stage *Pf*3D7. Parasites were incubated in 10× EC_50_ **MMV1557817** for either 24 or 48 h before the compound was washed off and parasites were allowed to grow for a further 48 h. Survival was determined via Sybr Green I assay and compared with vehicle (DMSO)-treated controls. Shown is the mean ± standard deviation (*n* = 4). Statistical significance was determined using a one-way ANOVA. (**D**) *Pf*3D7 viability time course profiles for **MMV1557817** compared with chloroquine. The lag phase is indicated on the graph and summarized in the table below, along with the log of the parasite reduction ratio (PRR) and the 99.9% parasite clearance time (PCT; *n* ≥ 2 performed in quadruple). (**E**) Killing action of **MMV1557817** and current antimalarial compounds against *ex vivo P. falciparum* (solid icons) and *P. vivax* (clear icons) obtained from clinical isolates. No significant differences in EC_50_ values were observed for these compounds between species, with the exception of chloroquine.

Preliminary characterization of **MMV1557817** against *P. falciparum* asexual *Pf*3D7 and Dd2 parasites was performed previously using an image-based screening assay (56) and the EC_50_ found to be in the low nanomolar range (11). To determine the effect of **MMV1557817** on parasite growth, we investigated the stage at which growth was affected after treatment. Using the EC_50_ determined (Fig. 2A; 39 nM, 31.8–46.9 CI), *Pf*3D7 cultures at 0–4 h old were treated with 5× or 10× the EC_50_. Both treatments resulted in stalling of parasite growth at the ring stage, while parasites treated with the 10× concentration of DMSO continued through the cycle before reinvading into the following cycle approx. 48 h after treatment began (Fig. 2B).

Wash-out experiments were performed to further analyze asexual killing, with artesunate serving as a positive control (EC_50_ 4 nM, 0.5–6.5 CI). Treatment with **MMV1557817** for 48 h, starting at the ring stage, was significantly more effective at killing parasites than a 24 h exposure. When treatment was initiated at the trophozoite stage, 24 h exposure to **MMV1557817** was significantly more effective than a similar exposure at the ring stage (Fig. 2C). With the exception of the 24 h treatment of rings, there was no significant difference between parasite survival after treatment with 10× EC_50_ of **MMV1557817** or artesunate for the different time periods (Fig. 2C).

Next, the PRR and PCT of **MMV1557817** were assessed *in vitro*, as both represent important mode-of-action parameters to determine the likelihood of parasite recrudescence and drug resistance *in vivo*. **MMV1557817** showed a lag phase of 72 h before reaching its optimal killing rate. After this lag phase, the PRR over one cycle was 2.2 ± 0.2 and the 99.9% PCT was 86 h (Fig. 2D). The antimalarial chloroquine was used as a positive control, with a PRR of 3.9 ± 0.2 and the 99.9% PCT of 53 h, similar to what has been described previously (37).

The ability of **MMV1557817** to kill the sexual stages of *P. falciparum* was also determined using image-based assays. The IC_50_ values for early (stages I–III; *n* = 3), late (stages IV–V; *n* = 3) and mature (stage V; *n* = 2) gametocytes were determined to be 99 nM, 309 nM, and 1474 nM, respectively (Table S2). While there is a reduction in the activity against the sexual stages compared with the asexual stages, **MMV1557817** is nevertheless still effective against early- and late-stage gametocytes at sub-micromolar concentrations.

### MMV1557817 retains nanomolar range activity against drug-resistant and clinical isolates of *P. falciparum* and *P. vivax*

To determine if there was cross resistance of **MMV1557817** against parasites harboring mutations that confer resistance to current antimalarials, activity was tested against a panel of drug-resistant field isolates using the previously described [^3^H]hypoxanthine incorporation assay (34) and compared with the CQ-sensitive field isolate NF54 (EC_50_ = 22 nM; Table 1). The strains that were tested include K1 (CQ, pyrimethamine, and sulfadoxine resistant), 7G8 (CQ resistant), TM90C2B (atovaquone resistant), Cam3.1 (artemisinin resistant) and Dd2 (CQ resistant; previously tested [11]). No shift in **MMV1557817** EC_50_ was observed when compared with the NF54 strain, indicating that the compound is not susceptible to any known resistance mutations tested here (Table 1). Importantly, no loss of efficacy was demonstrated for the artemisinin-resistant Cam3.1 strain, suggesting that the drug would be effective against artemisinin-resistant parasites. We additionally investigated the FIC of artemisinin and **MMV1557817** used in combination on Dd2 parasites to determine any potential interactions between the two compounds. The ΣFIC of each ratio combination found the compounds to function in an additive manner, whereby each compound retained their activity in the presence of the other (Fig. S1; Table S3). **MMV1557817** was further tested against laboratory-selected Dd2 parasites that harbored additional resistance mutations to current novel antimalarials including DDD107498 (protein synthesis inhibitor [57]], MMV390048 (phosphatidylinositol 4-kinase inhibitor [58]], DSM265 (dihydroorotate dehydrogenase inhibitor [59]), GNF156 (*Pf*carl [60]), and ELQ300 (cytochrome bc1 complex inhibitor [61]) and again showed no EC_50_ shift compared with the parent Dd2 line indicating that **MMV1557817** is not targeting these mechanisms.

**TABLE 1:** MMV1557817 effectiveness against drug-resistant *P. falciparum* strains^*a*^

Parasite strain	EC_50_ nM (mean)^*b*^	Fold shift EC_50_ relative to		Altered genes
		NF54	Dd2	
NF54	22	1.0		Sensitive strain
K1	26	1.2		*Pfcrt*, *Pfmdr1*, *Pfdhfr*, *Pfdhps*
7G8	28	1.3		*Pfcrt*, *Pfmdr1*, *Pfdhfr*, *Pfdhps*
TM90C2B	21	1.0		*Pfcrt*, *Pfmdr1*, *Pfdhfr*, *Pfdhps*, *Pfcytb*_Q0_
Cam3.1 (MRA1240)	23	1.1		*Pfcrt*, *Pfmdr1*, *Pfdhfr*, *Pfdhps*, *Pfkelch13*
Dd2	23	1.1	1.0	*Pf*crt, *Pfmdr1*, *Pfdhfr*, *Pfdhps*
Dd2 DDD107498	24		1.0	Dd2 + *Pfeef2*
Dd2 MMV390048	19		0.8	Dd2 + *Pfpi4*k
Dd2 DSM265	21		0.9	Dd2 + *Pfdhodh*
Dd2 GNF156	22		1.0	Dd2 + *Pfcarl*
Dd2 ELQ300	22		1.0	Dd2 + *PfcytB*

^*a*^ Seventy-two-hour [^3^H]hypoxanthine incorporation assay.

^*b*^ Mean values from two independent biological replicates.

To assess the efficacy of **MMV1557817** against clinical isolates, *ex vivo* susceptibility was performed on isolates from 23 patients presenting to malaria clinics in Timika (Indonesia) with single-species infections of either *P. falciparum* (*n* = 15) or *P. vivax* (*n* = 8) between January and April 2016. Susceptibility profiles in the same isolates were also determined for standard anti-malarials chloroquine, piperaquine, mefloquine, and artesunate. Adequate growth after harvesting was achieved for all (15/15) of *P. falciparum* isolates and 75% (6/8) of *P. vivax* isolates. Baseline characteristics of the isolates are presented in Table S4. Drug susceptibility did not differ significantly between species for the anti-malarial drugs piperaquine, mefloquine, and artesunate, as well as **MMV1557817** (median EC_50_: 98.1 nM for *P. falciparum* versus 68.6 nM for *P. vivax*; *P* = 0.533; Fig. 2E; Table S5). However, significantly greater activity was observed against *P. vivax* than *P. falciparum* for CQ (median EC_50_: 64.8 nM for *P. falciparum* versus 36.4 nM for *P. vivax*; *P* = 0.043). The nanomolar efficacy of **MMV1557817** against *P. vivax* confirms cross species killing consistent with previously generated *K*_i_ values against *P. falciparum* and *P. vivax* recombinant M1 and M17 proteins (11).

### MMV1557817 shows good selectivity, safety, ADME, and exposure properties and is active *in vivo* against the rodent malaria species *Plasmodium berghei*

Previously, cellular toxicity of **MMV1557817** was measured against HEK293 cells, where a selectivity index of ≥1,370 was seen at 10 µM (11). Additionally, selectivity of **MMV1557817** was assessed against MMP2, 7, 8, 9, and 13, as well as the human M1 aminopeptidase insulin-regulated aminopeptidase (IRAP) and the human aminopeptidase N (APN or CD13) (11). Collectively, these studies identified that **MMV1557817** shows limited off-target inhibitory affects against the MMPs and the IRAP (tested up to 200 µM). For the human APN, some cross-reactivity with a *K*_i_ of 0.3 µM was observed. Given this cross-reactivity with human APN, we further assessed the efficacy of **MMV1557817** toward other human M1 aminopeptidases of current or future therapeutic interest (62). Here, we tested human leukotriene A4 hydrolase (LTA4H) and the endoplasmic reticulum aminopeptidases 1 and 2 (ERAP1/2). These enzymes were purchased commercially and showed limited activity in our *in vitro* assay, and as such, an appropriate 12-point dose range in triplicate was not viable and a *K*_i_ calculation could not be completed. Thus, the percentage of enzyme activity was compared in the presence of different concentrations of **MMV1557817** or artesunate (Table S6). The results showed that at 1.25 µM compound, **MMV1557817** resulted in 18% inhibition of LTA4H but had no activity toward either ERAP. At the highest concentration tested (1 mM), there was near complete inhibition of all three human homologs. Artesunate showed minimal inhibition on the activities of the human enzymes but did show moderate, dose-dependent inhibition of the *Plasmodium* M1 enzymes. Our results suggest that there is some cross-reactivity to human homologs; however, the selectivity index between human and *Plasmodium* target remains high (50–100).

*In vitro* pharmacology safety assays conducted using the EuroFins CEREP panel were used to assess potential off-target activity within DNA repair pathways (namely, the human histidine deacetylases HDAC1, 2, 5, 7, 8, 9, and 10) and protease pathways (human matrix metalloproteases MMP2, 3, 7, 8, 9, and 14) (Table S7). Overall, the results only identified MMP-8 as being above the 50% threshold for inhibition by **MMV1557817** at 10 µM (52.5% inhibition), with no notable stimulation or inhibition of any of the receptors tested.

Studies to assess physicochemical properties, plasma stability, and metabolic stability were performed with **MMV1557817** prior to assessing the exposure profile in mice. As shown in Table S8, the compound has a moderate molecular weight and polar surface area, a moderately high log D_7.4_, and low to moderate aqueous solubility. It was reasonably stable in liver microsomes and cryopreserved hepatocytes and in mouse and human plasma. The compound was highly bound to both plasma proteins (98%–99% bound) and in the Albumax medium (84.5% bound) used for *in vitro* activity assessment. Correcting for the binding in Albumax medium gives an unbound EC_50_ value of 3.4 nM (based on 15.5% unbound of NF54 EC_50_ 22 nM; Table 1).

Studies with human and rat hepatocytes identified three main primary metabolites (Fig. S2) corresponding to an amide hydrolysis product (*m*/*z* 380, M-15), a hydroxamic reduction product (*m*/*z* 379, M-16), and a glucuronidation product (*m*/*z* 571, M+176). Authentic standards for these metabolites were not available, which precluded an accurate assessment of the relative abundance of each. However, on the basis of the peak area alone (data not shown) and with the assumption of similar response factors for all metabolites, the hydrolysis product and glucuronide appeared to be the predominant products in human hepatocytes, while the hydroxamic reduction product and the glucuronide were more prominent in rat hepatocytes.

**Fig 3:**
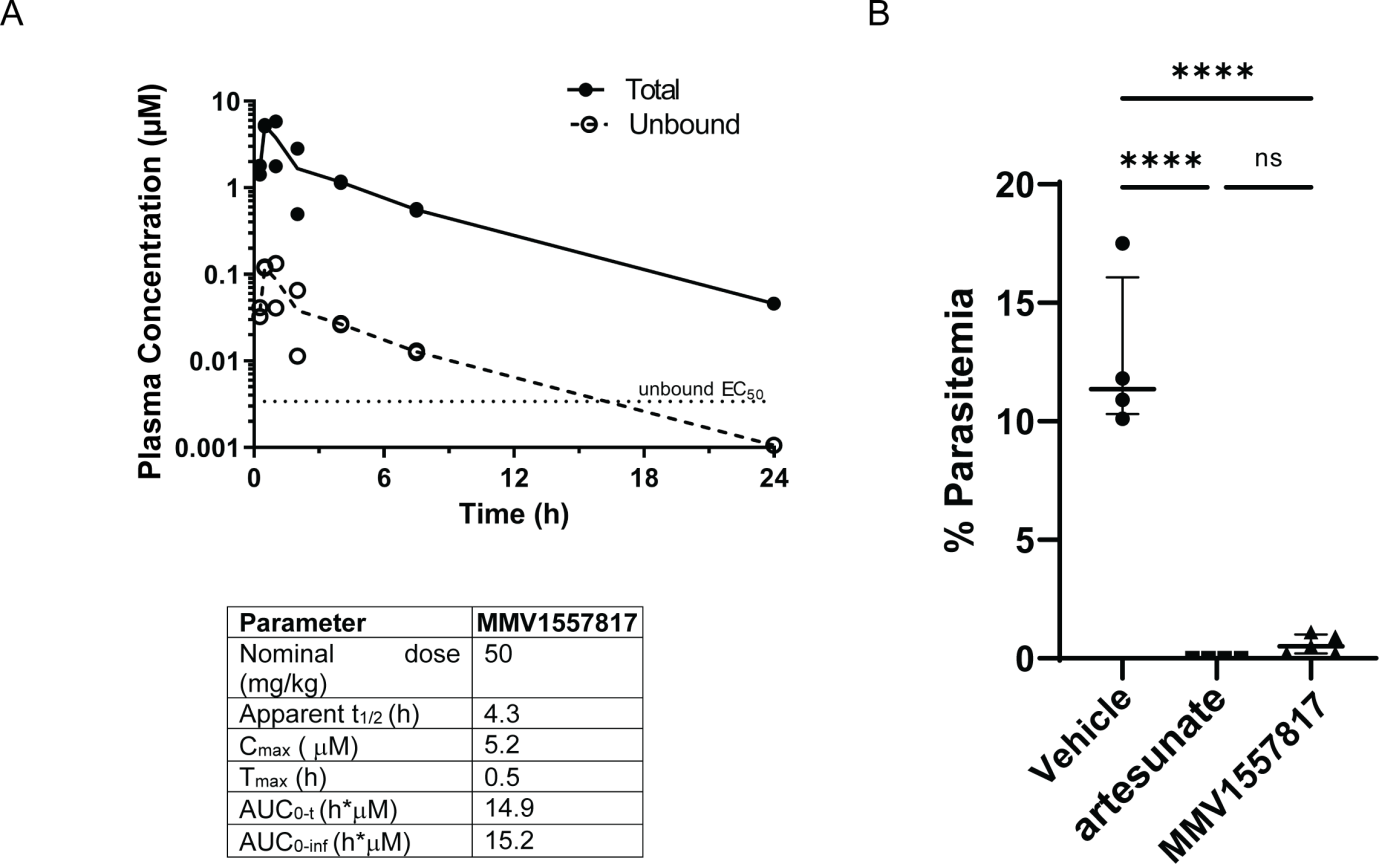
Bioavailability and activity of **MMV1557817** in murine models. (**A**) Upper panel: plasma concentration versus time profiles for **MMV1557817** following oral dosing (50 mg/kg) to non-infected male Swiss outbred mice. Concentrations are the total measured concentrations and calculated unbound concentrations (obtained by multiplying the total concentration by the fraction unbound in mouse plasma). The unbound *P. falciparum* EC_50_ value for **MMV1557817** is shown for comparison. Lower panel: plasma exposure parameters in Swiss outbred mice following oral administration. (**B**) Peter’s test performed on Balb/c mice infected with *P. berghei* ANKA parasites and treated with 50 mg/kg **MMV1557817** or 30 mg/kg artesunate on days 0, 1, 2, and 3 post-infection and parasite clearance determined on day 4. **MMV1557817** was found to clear 95.4% of infection. Significance was determined using an unpaired *t*-test; *n* ≥ 4.

Following this, uninfected mice were treated with a single oral dose of **MMV1557817** at 50 mg/kg administered in a suspension formulation and the plasma concentration versus time profiles as well as plasma exposure parameters were determined (Fig. 3A). Unbound concentrations were calculated using the measured free fraction in mouse plasma (Table S8). The half-life was found to be 4.3 h, with the overall results indicating that daily administration at this dose level would be expected to maintain unbound concentrations above the unbound EC_50_ (3.4 nM based on 15.5% unbound of NF54 EC_50_ 22 nM; Table 1) for approximately 14 h.

Given the good selectively and reasonable PK properties of **MMV1557817** and after confirming the effectiveness of the compound against recombinant *Pb*-M1 and *Pb*-M17 (Fig. 1A), the efficacy of **MMV1557817** was also tested in a preliminary *in vivo* model of malaria infection using the *P. berghei* ANKA rodent malaria 4-day suppressive test (48). Accordingly, 50 mg/kg **MMV1557817** or 30 mg/kg artesunate (positive control) was given by oral gavage (same formulation as used for the mouse exposure study) on days 0, 1, 2, and 3 post-infection, with parasite survival calculated on day 4 as per standard Peters’ test. **MMV1557817** showed excellent efficacy, with mice treated with this compound exhibiting a 95.4% parasite reduction when compared with the vehicle control, with no significant difference seen between **MMV1557817** treatment and the artesunate control (Fig. 3B).

### Thermal proteomics profiling and metabolomics confirms MMV1557817 targets both *Pf*A-M1 and *Pf*A-M17 aminopeptidases

**Fig 4:**
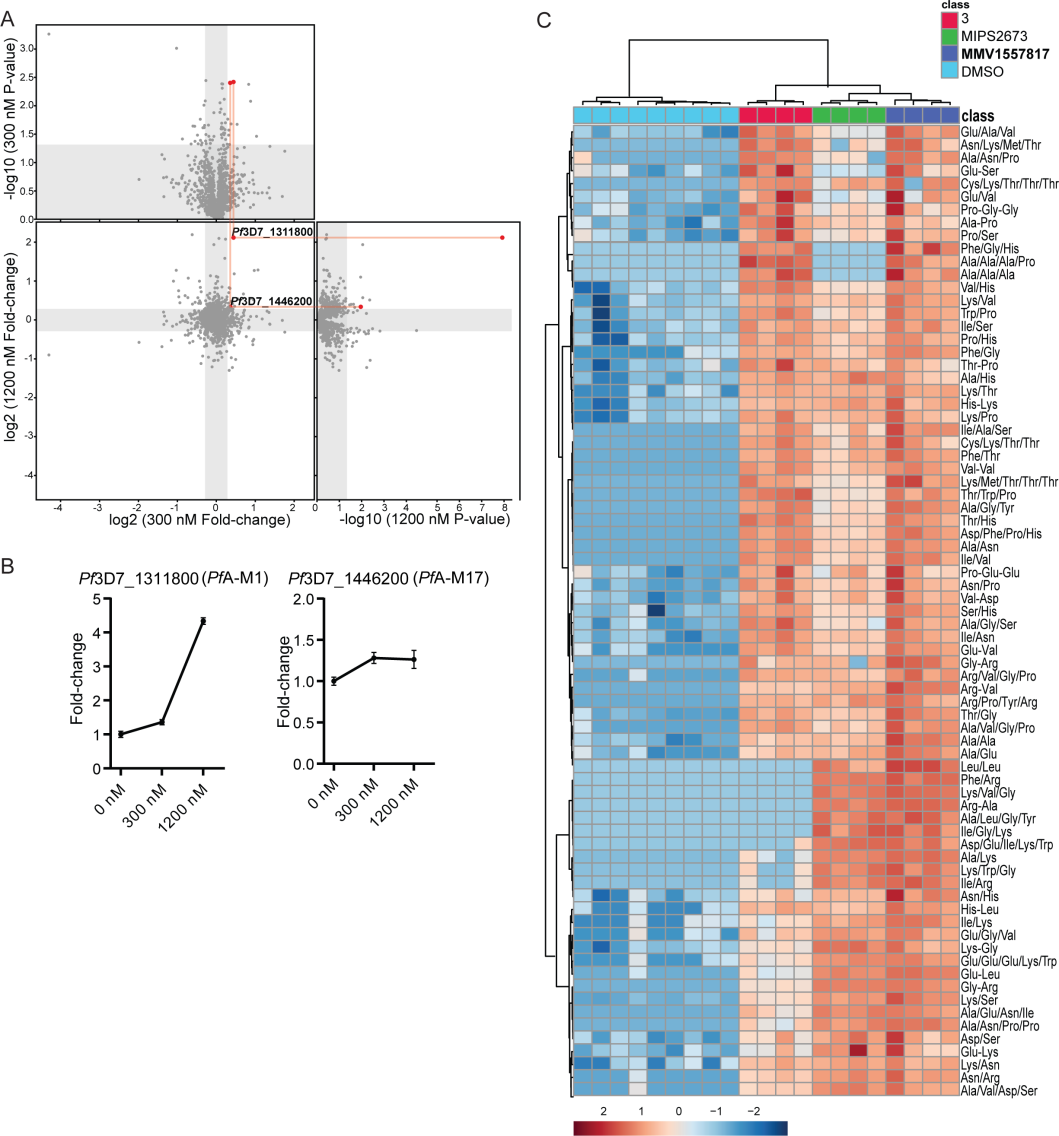
Thermal proteome profiling of **MMV1557817** confirms *Pf*A-M1 and *Pf*A-M17 as compound targets. (**A**) Paired volcano plot of all proteins detected. The outside panels show the log_2_ fold change vs –log_10_ *P* value of proteins following treatment with 300 nM or 1200 nM of **MMV1557817**, relative to the 0 µM negative control, following a 60°C thermal challenge. The thermal stability of both *Pf*A-M1 (*Pf*3D7_1311808) and *Pf*A-M17 (*Pf*3D7_1446200) were altered at both concentrations with a *P* value < 0.05 as determined by Welch’s *t*-test, with increasing stability in increasing concentrations. *Pf*A-M1 and *Pf*A-M17 are highlighted with red lines and dots. No other proteins were significantly stabilized by both drug concentrations. (**B**) Protein intensity of *Pf*3D7_1311808 (*Pf*A-M1) and *Pf*3D7_1446200 (*Pf*A-M17) in increasing concentration of **MMV1557817** and following 60°C thermal challenge. Value represents the mean of two biological replicates performed with ≥3 technical replicates ± standard deviation. (**C**) Hierarchical clustering of the 73 significantly perturbed peptides (*P* value < 0.05) following treatment with **MMV1557817**, MIPS2673 (*Pf*A-M1 inhibitor [53]), **3** (*Pf*A-M17 inhibitor [7]), and DMSO control. Vertical clustering displays similarities between samples, while horizontal clusters reveal the relative abundances of the 73 peptides from four to seven biological replicates. The color scale bar represents log2 (mean-centered and divided by the standard deviation of each variable) intensity values. Peptides with hyphen notations indicate confirmed sequence by MS/MS. Peptides with slash notation indicate putative amino acid composition (accurate mass), without confirmed sequence order.

**MMV1557817** shows aminopeptidase inhibition of both *Pf*A-M1 and *Pf*A-M17 in recombinant systems. To elucidate any other potential targets of **MMV1557817** within parasites, thermal proteomics profiling (TPP) was utilized. TPP is based on the principle that, when heated, parasite proteins will denature and can be removed by ultracentrifugation (100,000 *g*) due to their insolubility. In contrast, any protein bound to **MMV1557817** will exhibit enhanced thermal stability, protecting it from the thermal challenge and resulting in an increased concentration of protein in the soluble fraction, which can be identified by LC-MS-based proteomics analysis. Two TPP experiments were performed with three to four technical replicates and a thermal challenge of a single temperature at 60°C with low (300 nM [10× EC_50_], experiment 1]) and high [1,200 nM [40× EC_50_], experiment 2) compound concentrations. Common proteins (1,405) between experiment 1 (1,586) and experiment 2 (1,853) identified by LC-MS/MS were analyzed, and only two proteins had altered thermal stability profiles (fold change > 1.2 and *P* value < 0.05) across the two concentrations of **MMV1557817** compared with the DMSO vehicle-treated lysates (0 nM; Fig. 4A). These proteins were *Pf*A-M1 (*PF*3D7_1311800) and *Pf*A-M17 (*Pf*3D7_1446200). *Pf*A-M1 demonstrated a 1.36-fold stabilization following incubation with 300 nM **MMV1557817** and a 4.33-fold stabilization with 1,200 nM compared with 0 nM. *Pf*A-M17 demonstrated a significant 1.28-fold stabilization with 300 nM **MMV1557817** treatment and 1.26-fold stabilization with 1,200 nM compared with 0 nM (Fig. 4B). This independent and unbiased drug target identification approach identified *Pf*A-M1 and *Pf*A-M17 as the likely protein targets of **MMV1557817**.

To further confirm that **MMV1557817** targets *Pf*A-M1 and *Pf*A-M17, we compared metabolomics profiles of *Pf*3D7 parasites treated with 10× EC_50_ of **MMV1557817**, MIPS2673 (*Pf*A-M1 inhibitor [53]), **3** (*Pf*A-M17 inhibitor [7]), or DMSO control (Fig. S3). Principal component analysis and heatmap analysis of relative abundances of putative metabolites dysregulated following treatment with the inhibitors demonstrated that the most prominent metabolomic signature shared between them was a series of peptides that were increased (Fig. 4C; Fig. S3). Further detailed analysis of dysregulated peptides shared among the inhibitors demonstrates that **MMV1557817** increases the levels of peptides that also increase in abundance following specific inhibition *Pf*A-M1 and *Pf*A-M17 (Fig. 4C), consistent with additive inhibition of both aminopeptidases. We have previously shown that majority of these peptides could be mapped to hemoglobin sequences (7, 53).

### A single nucleotide polymorphism in *Pf*A-M17 identified in MMV1557817-resistant parasites impacts enzyme hexamerization

While the targets of **MMV1557817** were confirmed using the above methods, it is also important to assess the ability of parasites to become resistant to the compound and the potential impact this has on other antimalarial drugs. Recent *in vitro* resistance selection and whole genome analysis undertaken to determine if resistance against **MMV1557817** could be selected in Dd2 parasites identified a A460S mutation in one of its intended targets, *Pf*A-M17, as well as a N846I mutation in an AP-3 β subunit (*PF*3D7_0613500) and a M317I mutation in a non-essential conserved *Plasmodium* protein (*PF*3D7_1144400) (63). These three mutations were present in all clones obtained from multiple flasks, with parasites only displaying modest EC_50_ shifts of between 1.5× and 2.9×, suggesting a low level of resistance (63). To confirm whether the A460S mutation was responsible for resistance, CRISPR/Cas9 technology was utilized to attempt to introduce this mutation into *Pf*A-M17 in *Pf*3D7 wild-type parasites using a previously described donor plasmid method (64). However, after 4 weeks, no mutants could be generated despite a silent mutation being incorporated into *Pf*A-M17 within this time (gct to gcc; Fig. S4) using the same guide RNA. This suggests that the A460S mutation may not be viable in parasites without the other compensatory mutations found in the *in vitro* resistance selection studies or that parasite survival is limited by the apparent fitness cost seen in **MMV1557817**-resistant parasites harbouring this mutation (Fig. S5).

**Fig 5:**
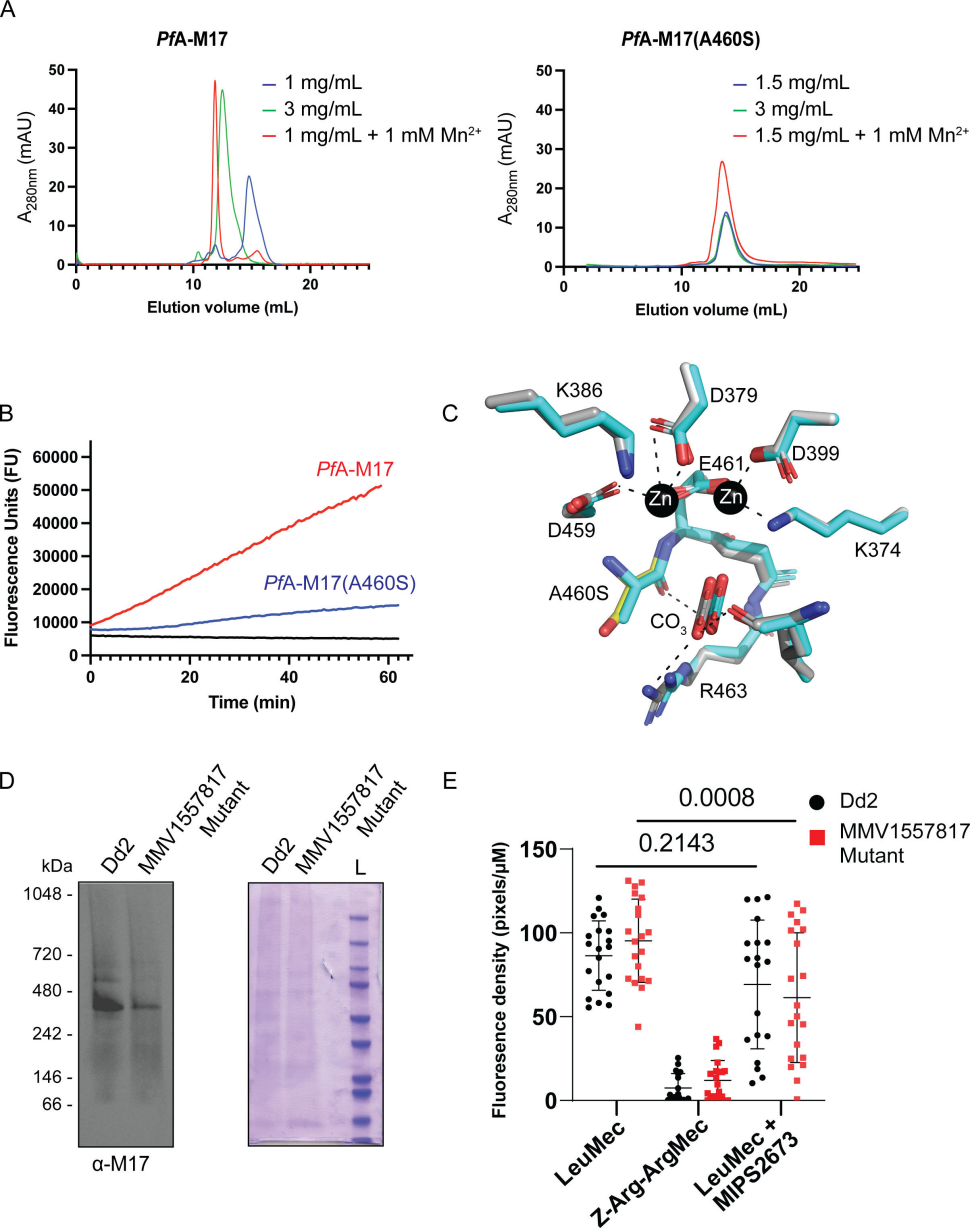
Characterization of *Pf*A-M17(A460S). (**A**) Size exclusion chromatography of recombinant wild-type *Pf*A-M17 (left) and mutant *Pf*A-M17(A460S) (right) showing that the shift to the functional hexameric species does not occur in the mutant as readily as wild-type. Concentration of recombinant proteins analyzed is indicated, as is the presence of metal ions that has been shown to shift the oligomeric equilibrium. (**B**) Activity assays comparing recombinant wild-type (red) at 300 nM to A460S mutant (blue) at 10 µM. Aminopeptidase assay is indicated by an increase in fluorescence units (*x*-axis) over time (*y*-axis). (**C**) X-ray crystal structure of the active site of A460S (grey sticks) shows little change compared with wild-type (cyan sticks) in zinc-coordinating residues nor interactions with the carbonate ion (labeled), required for activity. The mutation position S460 is shown as a yellow stick. Interactions are shown in dashed lines. (**D**) Western blot analysis of blue-native PAGE performed on trophozoite stage Dd2 or **MMV1557817**-resistant parasites solubilized in 0.25% Triton X-100 reveals the presence of a *Pf*A-M17-specific species representative of the native homohexamer (left panel). The expected size of the hexamer is 408 kDa, and each lane contains 10 µg of protein. Protein loading was confirmed by Coomassie stain (right panel). (**E**) Quantification of fluorescent density of proteolytic cleavage by *Pf*A-M1 and *Pf*A-M17 in Dd2 or **MMV1557817**-resistant parasites via live cell imaging with or without *Pf*A-M1 inhibition by MIPS2671. Z-Arg-ArgMec serves as a negative control. Plotted is the mean ± standard deviation, and significance was determined using a one-way ANOVA Dunnett’s test.

To assess the effect of the A460S single nucleotide polymorphism (SNP) on *Pf*A-M17 aminopeptidase activity, we introduced the same mutation into our recombinant gene expression construct and produced the protein *Pf*A-M17(A460S). The mutant protein was purified as per the same protocol used to generate wild-type protein, but it exhibited an altered behavior during the size exclusion chromatography step, suggesting that the mutation may have affected the ability of the protein to form the biologically active hexamer (16). Attempts to induce hexamer formation via metal supplementation failed to shift the oligomeric equilibrium toward the functional complex as readily as the wild-type (Fig. 5A). Assessment of enzymatic activity confirmed this result with increased concentrations of *Pf*A-M17(A460S) required for observable aminopeptidase activity (Fig. 5B). To assess if the mutant could form a hexamer, we solved the X-ray crystal structure of the recombinant *Pf*A-M17 (A460S) (Table S1). The 2.6 Å structure confirmed the protein can form a hexamer in the crystal lattice and suggests the lack of activity in solution may arise from an instability of the hexamer or oligomeric intermediates. Overall, the structure was virtually unchanged from the wild-type (0.351 root mean square deviation over 497 Cα atoms within the A-chain; Fig. 5C which shows the active site) and no gross changes in secondary or tertiary structure were observed. Further, a similar domain arrangement and no large regions of disorder were observed in electron density that were not already known to be flexible in wild-type structures. The location of residue A460 is in the active site, directly between two highly conserved zinc-coordinating residues, D459 and E461. Both D459 and E461 are involved in metal-protein interactions, and E461 shows bidentate zinc coordination when both metal sites are occupied (18). Interestingly, E461 has been shown to be essential for correct hexamerization and subsequent aminopeptidase activity of *Pf*A-M17 (16). The residue A460 forms a hydrogen bond with the carbonate ion, also present in the active site and required for proteolysis, but the bond is formed with the backbone carbonyl and, as such, is maintained when the residue is changed to A460S. The small side-chain A > S change only introduces a hydroxyl group; however, the sidechain does reach into a pocket that is largely hydrophobic. Whether this is enough to destabilize the folding of the pocket and active site or whether the change interferes with zinc binding (and hence destabilizes hexamer formation) remain unclear.

We next compared *Pf*A-M17 in lysates made from wild-type and **MMV1557817**-resistant parasites to assess hexamerization of the protein within parasites. Blue native PAGE confirmed that while *Pf*A-M17(A460S) could form hexamers in parasites, reduced amounts of hexamer was observed when equal amounts of protein was loaded (Fig. 5D). Further, live cell microscopy was employed to investigate the activity of *Pf*A-M17(A460S) in these resistant parasites. The fluorogenic peptide substrate L-leucine-7-amido-4-methylcoumarin hydrochloride (H-Leu-NHMec) can be cleaved by both *Pf*A-M1 and *Pf*A-M17 (54), with both Dd2 and **MMV1557817**-resistant parasites confirmed to cleave this substrate (Fig. 5E; Fig. S6). The peptide Z-Arg-Arg-7-amido-4-methylcoumarin hydrochloride (Z-Arg-ArgMec) is a N-terminally blocked substrate that is unable to be cleaved and serves as a background fluorescence control. Employing the previously published *Pf*A-M1-specific inhibitor MIPS2673 (53), parasites were treated with 10× the EC_50_ (3.2 µM) for 20 min to determine the level of residual *Pf*A-M17 function. Only **MMV1557817**-resistant parasites showed significantly reduced fluorescence with this treatment, suggesting that there is reduced active *Pf*A-M17 present in these parasites, possibly due to the destabilization of the homohexamer (Fig. 5E). This mechanism of resistance is likely to be limited by the essentiality of *Pf*A-M17 (7).

### MMV1557817-resistant parasites show altered susceptibility to aminopeptidase inhibitors and an increase in hemoglobin digestion

**Fig 6:**
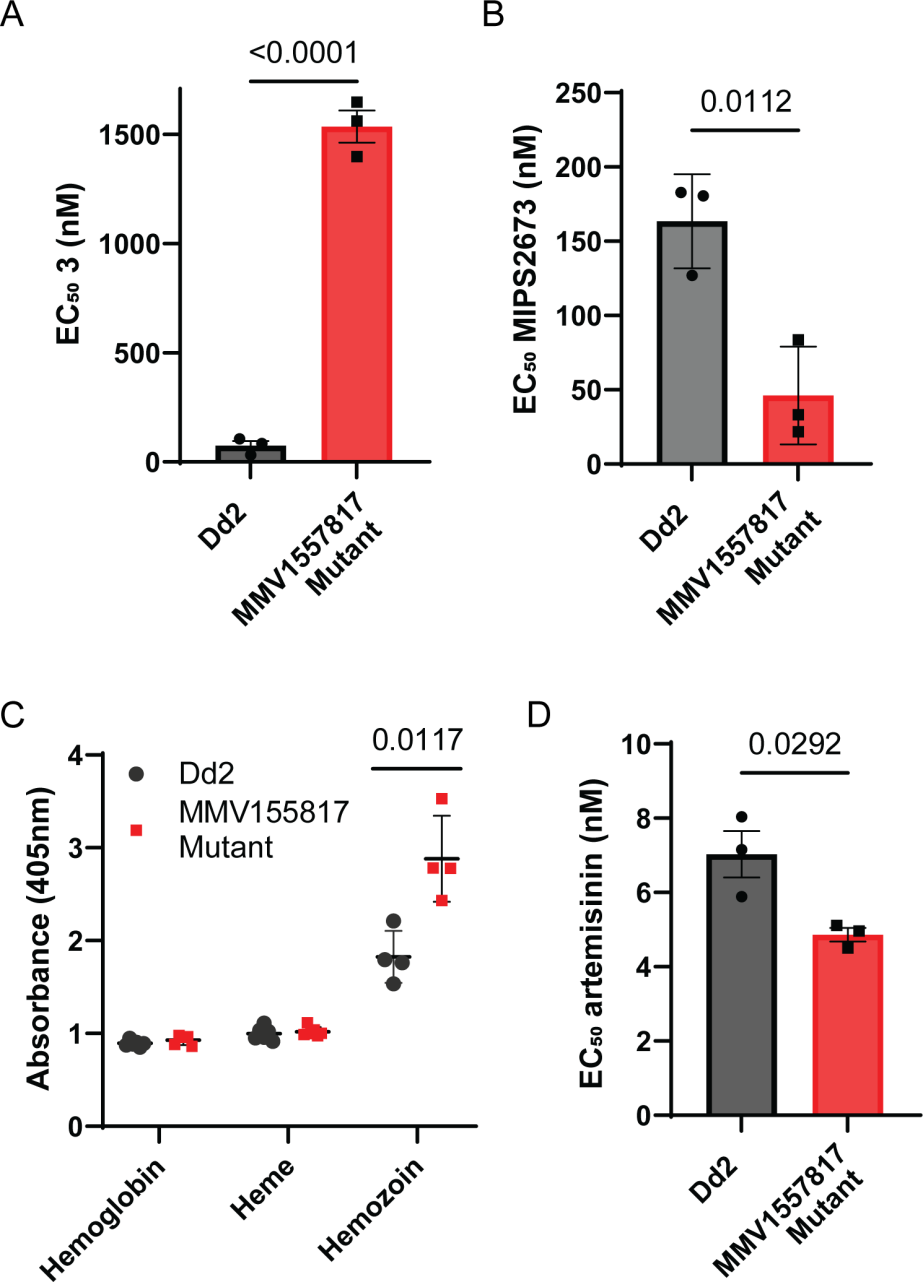
**MMV1557817**-resistant parasites show altered susceptibility to aminopeptidase inhibitors and artemisinin as well as an increase in hemoglobin digestion. Killing action of a (**A**) *Pf*A-M17-specific inhibitor (**3**) or (**B**) *Pf*A-M1-specific inhibitor (MIPS2673) shows altered EC_50_ values compared with parental Dd2 parasites from three biological replicates performed in triplicate and plotted as the mean ± standard deviation; significance determined by unpaired *t*-test. (**C**) Absorbance readings at 405 nm for hemoglobin, haem, and hemozoin species from 5.18 × 10^7^ Dd2 or **MMV1557817**-resistant parasites at mid-late trophozoite stage. Plotted is the mean ± standard deviation from ≥4 biological replicates; significance determined by Welch’s *t*-test. (**D**) Killing action of artemisinin showed significantly decreased EC_50_ values for the resistant strain compared with parental Dd2 parasites from three biological replicates performed in triplicate and plotted as the mean ± standard deviation; significance determined by unpaired *t*-test.

To further assess the effect of the SNPs on **MMV1557817**-resistant parasites, including the A460S mutation in *Pf*A-M17, we measured the parasite killing of individual aminopeptidase inhibitors against these parasites. The EC_50_ values were determined using the resistant parasites treated with either the *Pf*A-M17-specific inhibitor compound **3** (7) or the *Pf*A-M1-specific inhibitor MIPS2673 (53). Parasites were found to be resistant to the *Pf*A-M17 specific inhibitor, with the EC_50_ showing an increase of 22× when compared with the parental Dd2 parasites (Fig. 6A). **MMV1557817**-resistant parasites were sensitized to the specific *Pf*A-M1 inhibitor, showing an approx. 3.5× decrease in EC_50_ (Fig. 6B). Considering that both enzymes are essential for providing parasites with amino acids from hemoglobin, a decrease in *Pf*A-M17 possibly results in a greater reliance on *Pf*A-M1 for digestion, therefore sensitizing parasites to this specific inhibitor.

Considering these changes to aminopeptidase inhibition, as well as the potential impact of the A460S mutation on *Pf*A-M17, we next investigated if there were alterations in hemoglobin digestion in the resistant parasites through hemoglobin fractionation. The quantities of hemoglobin, haem, and hemozoin in resistant parasites were compared with the parental Dd2 line at the mid-late trophozoite stage (Fig. 6C). While no changes were seen for hemoglobin or haem, there was a significant increase in hemozoin in resistant parasites, with parasites harboring 1.5× more compared with the Dd2 line (Fig. 6C). As the majority of hemoglobin digestion is likely to have already occurred at this later stage of the parasite lifecycle, this increase suggests that there is likely an overall increase in hemoglobin digestion in resistant parasites. Given this, we measured the antiparasitic activity of artemisinin as it requires hemoglobin digestion for drug activation. **MMV1557817**-resistant parasites were found to be marginally but significantly sensitized to artemisinin in a 72 h killing assay (Fig. 6D).

## DISCUSSION

As front-line antimalarial treatments continue to be threatened by drug resistance, it is paramount that development of alternative drugs is maintained. Here, we show that M1 and M17 inhibitors are excellent candidates for new antimalarial compound development with novel mechanisms of action. We extensively characterized the dual M1 and M17 aminopeptidase inhibitor, **MMV1557817**, which shows potent, on-target activity, using a range of recombinant- and parasite-based assays. The compound showed nanomolar activity toward both *ex vivo P. falciparum* and *P. vivax* parasites, confirming its cross-species activity, as well as a low-nanomolar killing range against drug-resistant parasites, confirming there is no cross resistance with current known mechanisms. The compound did show selectivity over human analogs; however, improvement of the magnitude of selectivity over human APN would be desirable but not considered essential given the ability to inhibit this human enzyme for long treatment periods with little to no known toxicity (65). **MMV1557817** was safely administered in a murine model of malaria, where it was able to show excellent activity in the rodent malaria 4-day *in vivo* suppressive test. Promisingly, **MMV1557817** shows a low-resistance liability, with resistant parasites marginally sensitized to artemisinin, likely due to an increase in hemoglobin digestion. It did however have a delay to reaching maximal parasite killing, as well as a relatively short plasma half-life, both of which are important aspects to consider in future medicinal chemistry development of inhibitors from this series. Overall, these results suggest that M1 and M17 inhibitors such as **MMV1557817** are worthy of further investigation as potential new antimalarial treatments.

Multiple independent approaches verified that **MMV1557817** targets both *P. falciparum* M1 and M17. Previous studies identified *Pf*A-M17 as a major molecular target for **MMV1557817** (56). We extend this finding using TPP, which confirms both *Pf*A-M1 and *Pf*A-M17 are bound by the compound, stabilizing these targets at 10–40 times their EC_50_ value. Metabolomics analysis of parasites treated with this dual *Pf*A-M1 and *Pf*A-M17 inhibitor found the profile of increased peptides to be a combination of the profiles previously seen in individual enzyme inhibition (7, 53). Taken together, this shows that the primary molecular targets of **MM1557817** are both *Pf*A-M1 and *Pf*A-M17. Resistant parasites generated against **MMV1557817** failed to identify any mutation in *Pf*A-M1, but analysis of the compound’s inhibitory activity may also provide an explanation as to why resistance studies did not isolate a mutation in *Pf*A-M1. In recombinant enzyme assays, **MMV1557817** is two-fold more potent toward *Pf*A-M17 than *Pf*A-M1 (11). The *in vitro* resistance selection in parasites was performed at 90 nM (63), which is similar to the *Pf*A-M17 *K*_i_ (112 nM) but still not as high as the *Pf*A-M1 *K*_i_ (308 nM). In contrast, the TPP was performed at 300 and 1,200 nM, both concentrations high enough to bind *Pf*A-M1. We hypothesize that this is the likely explanation for the discrepancy between resistance studies and TPP target identification. Development of **MMV1557817** resistance also only resulted in a very low shift in EC_50_ (1.5–2.9×), as well as a significant growth phenotype, suggesting that resistance is not easy to generate, possibly due to the presence of dual targets within the parasite.

The SNP in *Pf*A-M17 identified in **MMV1557817**-resistant parasites alludes to an intriguing mechanism of resistance. Analysis of recombinant *Pf*A-M17 harboring the A460S mutation suggests that the ability of the protein to form a stable hexamer—essential for aminopeptidase activity—was affected. This also appeared to be the case in resistant parasites, which harbored less *Pf*A-M17 hexamer and showed reduced enzymatic activity. Given that *Pf*A-M17 is essential for parasite survival (7), this mode of resistance is unlikely to lead to full destabilization of the hexamer as this would likely result in parasite death. Alongside the considerable fitness cost seen in these parasites, it is unlikely that they could persist in an endemic population. Comparison of the binding mode of **MMV1557817** and the active site of *Pf*A-M17(A460S) protein suggests that inhibitor binding would not be affected but inhibition could not be assessed due to the lack of activity observed in *in vitro* assays. However, a single A460S mutation in *Pf*A-M17 could also not be reintroduced into *Pf*3D7 parasites, suggesting that the other two SNPs identified may be compensating for a catalytically compromised *Pf*A-M17(A460S) in resistant parasites. While one additional mutation was found in an unknown, non-essential *P. falciparum* protein, the other was identified in the AP-3 β subunit, potentially providing a further layer to **MMV1557817** resistance. This gene is currently only putatively annotated but appears to be essential (20). In mammalian and plant cells, adaptor protein-3 homologs have been shown to be involved in both clathrin-dependent and independent vesicle formation for organelles such as lysosomes, as well as protein cargo sorting in relation to the endoplasmic reticulum and the Golgi apparatus (66–68). The digestive vacuole is analogous to a lysosome, and indeed, other adaptor proteins have been identified in the hemoglobin digestion pathway, with AP-3 a notable omission (69). Further investigation into the involvement of the AP-3β subunit in **MMV1557817** resistance may lead to further information on protein trafficking, formation of the digestive vacuole, and hemoglobin digestion, where it potentially is responsible for the increase in hemoglobin digestion seen in these parasites.

**MMV1557817** resistance also altered the susceptibility of parasites to individual *Pf*A-M1 and *Pf*A-M17 inhibitors. The *Pf*A-M17-specific inhibitor exhibited a 22× increase in the EC_50_ value in the resistant line when compared with the parental line, reflecting that its target—a functional *Pf*A-M17 hexamer—was not present or present at a reduced concentration compared with wild type, in keeping with the mechanism of resistance. While the same amount of target is present for compound **3** to bind, it is no longer all in a functional form and as such results in resistance to a *Pf*A-M17-only inhibitor, highlighting the significance of **MMV1557817** as a dual inhibitor. The **MMV1557817**-resistant parasites also showed an apparent reliance on *Pf*A-M1, seen by the sensitization to the *Pf*A-M1-specific inhibitor. Although these aminopeptidases have been shown to have different substrate preferences (15), *Pf*A-M1 is able to cleave a subset of *Pf*A-M17 residues but cannot account for its complete loss (7). Additionally, loss of *Pf*A-M17 has not previously been shown to result in a build-up of apparent *Pf*A-M17-specific peptides but rather an overall increase in hemoglobin-derived peptides (7). This suggests that any decrease in functional *Pf*A-M17 likely results in an overall perturbation in peptide digestion which may account for the *Pf*A-M1 sensitization seen in **MMV1557817**-resistant parasites. This could also be the driving factor for an overall increase in hemoglobin digestion in an attempt by the parasites to increase the free amino acid pool to overcome partial loss of *Pf*A-M17.

**MMV1557817**-resistant parasites were also found to be marginally sensitized to artemisinin in the EC_50_ assay performed here. While this small degree of sensitization is unlikely to be clinically relevant, it suggests that inhibition of end-stage hemoglobin digestion is an attractive combination for artemisinin and its derivatives, with resistance to either compound less likely if used together. Whether this sensitization is due to the increase in hemoglobin digestion in these parasites or another stressor is yet to be determined. It could, however, be expected that an increase in hemoglobin digestion may also increase the potency of other antimalarial compounds targeting this pathway. For example, chloroquine and its derivatives cause parasite death by blocking the detoxification of haem (70), and as such, more hemoglobin digestion may influence this drugs’ efficacy. Interestingly, piperaquine-resistant parasites were also sensitized to dual aminopeptidase inhibition by **MMV1557817** (63). These parasites digest significantly less hemoglobin, highlighting the overall relationship of **MMV1557817**’s activity with hemoglobin digestion and suggesting inhibition of *Pf*A-M1 and *Pf*A-M17 could be useful in a number of different combination therapies. The parasite reduction ratio was however found to be modest, with a parasite clearance time of 86 h likely due to the 72 h lag phase before maximal killing occurs. These killing parameters are not dissimilar to atovaquone, which also has a considerable lag time until maximal killing occurs and a 99.9% parasite clearance time greater than 3 days (37). However, considering that washout experiments were performed after only 24 or 48 h of treatment and resulted in less than 10% parasite survival when parasites traversed a trophozoite stage, this lag phase is unlikely to be detrimental, particularly if used in partnership with other fast-acting drugs or developed further.

**MMV1557817** was also found to be effective against *P. falciparum* gametocytes at sub-micromolar concentrations. Activity was noticeably reduced as the parasites matured to later stage/mature gametocytes, which is not surprising, consistent with this compound targeting aminopeptidases that function in hemoglobin digestion which becomes less prominent as the sexual stages mature. It remains to be seen whether **MMV1557817** is active against other stages of the *P. falciparum* lifecycle, but it appears that *Pf*A-M1 and *Pf*A-M17 are transcribed in the mosquito stage of the lifecycle (71). *Pf*A-M1 and *Pf*A-M17 digest proteins that originate from sources in addition to hemoglobin (7, 53) and as such could be active throughout the entire lifecycle, making them an excellent antimalarial target. Of significance, **MMV1557817** showed activity against both *P. falciparum* and *P. vivax ex vivo* parasites within a nanomolar range. There was also a trend toward the compound being more effective against *P. vivax*; however, this did not reach significance (*P* = 0.0533). Recombinant assays highlight that **MMV1557817** is most potent against *Pv*-M1, with a *K*_i_ of 19 nM, which is likely contributing to its lower EC_50_ value against *ex vivo P. vivax* compared with *P. falciparum*. An antimalarial compound that has cross-species activity is highly desirable.

The safety profile of **MMV1557817** was explored against human M1 aminopeptidases to assess the likelihood of off-target effects. Human LTA4H, ERAP1, and ERAP2 were not found to be significantly inhibited at biologically relevant concentrations of **MMV1557817**, and neither were several HDAC enzymes, suggesting that despite the hydroxamic acid group’s ability to bind metal ions, there are no apparent widespread issues with off-target enzyme activity. Exposure in mice indicated a modest half-life of 4.3 h; however, further PK studies to determine PK parameters and oral bioavailability were not conducted. At an oral dose of 50 mg/kg in mice, unbound concentrations of **MMV1557817** were likely present above the unbound EC_50_ for about 14 h. Despite this limited exposure profile, inhibition of aminopeptidases in the rodent malaria model *P. berghei* was sufficient to substantially reduce parasitemia, with no significant difference seen when compared with the artesunate-positive control. For future development of drug leads, additional studies should be performed to assess long-term efficacy, including in humanized mouse models given **MMV1557817** was shown to be more active against recombinant *P. berghei* aminopeptidases. Interestingly, *Pb*-M17 is not essential for parasite survival; however, knockout of the gene results in a slow growth phenotype (72). Treatment with a dual M1 and M17 inhibitor of this potency is evidently effective, possibly due to the essentiality of *Pb*-M1 coupled with the very low *K*_i_ inhibition constant for this enzyme.

In conclusion, we have characterized a novel on-target aminopeptidase inhibitor that kills multiple *Plasmodium* species and found it to be a candidate for development as a lead antimalarial compound. **MMV1557817** was confirmed to be active against multiple stages of *P. falciparum* and encouragingly also kills *P. vivax*, an often neglected species for antimalarial drug development. Resistance to **MMV1557817** resulted in destabilization of the *Pf*A-M17 hexamer which is required for enzymatic function and is likely limited by the essentiality of this protein. Indeed, **MMV1557817** has proven to be a useful and effective tool molecule in validating the strategy of developing dual M1 and M17 inhibitors as antimalarial agents, while further optimization is clearly required to produce a drug candidate. More specifically, it would be beneficial to attenuate the metabolic hotspots present in **MMV1557817** to obtain longer-acting agents which would likely address key criteria outlined in the target product profiles set for new antimalarial drugs by the Medicines for Malaria Venture. To date, the success in employing a structure-based design to obtain potent inhibitors f both the M1 and M17 enzymes provides every confidence that dual inhibitory activity can be maintained throughout any future lead optimization studies. Overall, these results suggest that dual-acting M1 and M17 aminopeptidase inhibitors such as **MMV1557817** are worthy of further investigation as potential new antimalarial treatments.

## Data Availability

The mass spectrometry proteomics data haves been deposited to the ProteomeXchange Consortium via the PRIDE partner repository with the data set identifier PXD046006.

## SUPPLEMENTAL MATERIAL

Supplemental Data mbio.00966-24-s0001.pdf Supplemental tables and figures.

ASM does not own the copyrights to Supplemental Material that may be linked to, or accessed through, an article. The authors have granted ASM a non-exclusive, world-wide license to publish the Supplemental Material files. Please contact the corresponding author directly for reuse.
