# Supplementary material for: On-target, dual aminopeptidase inhibition provides cross-species antimalarial activity
Source: mBio. 2024 May 8;15(6):e00966-24. doi: 10.1128/mbio.00966-24 (PMC11237774; doi:10.1128/mbio.00966-24)
Supplement: Supplemental Data — Supplemental tables and figures. [file mbio.00966-24-s0001.pdf]

On-target, dual aminopeptidase inhibition provides cross-species antimalarial activity

Rebecca C.S. Edgar[1,2], Tess R. Malcolm[3], Ghizal Siddiqui[4], Carlo Giannangelo[4], Natalie A. Counihan[1,2], Matthew Challis[4], Sandra Duffy[5], Mrittika Chowdhury[1,2], Jutta Marfurt[6], Madeline Dans[1,2], Grennady Wirjanata[6], Rintis Noviyanti[7], Kajal Daware[3], Chathura D. Suraweera[3], Ric N Price[6,8,9], Sergio Wittlin[10,11], Vicky M. Avery[12], Nyssa Drinkwater[3], Susan A. Charman[13], Darren J. Creek[4], Tania F. de Koning-Ward[1,2#], Peter J. Scammells[14#] & Sheena McGowan[3#]

SUPPLEMENTARY DATA

Supplementary Table 1: Data collection and refinement statistics for X-ray crystal structures

	*Pf*A-M1 - **MMV1557817**	*Pf*A-M17 - **MMV1557817**	*Pf*A-M17(A460S)
PDB ID	8SVL	8SVM	8SW9
Data Collection			
Wavelength	0.95370	0.95370	0.95370
Resolution range	41.29 - 1.504 (1.56 - 1.50)	46.5 - 2.3 (2.382 - 2.3)	49.57 - 2.6 (2.69 - 2.60)
Space group	P 21 21 21	P 21 21 21	P 21 21 21
Unit cell (a, b, c, α, β, γ)	74.90 109.01 118.03 90 90 90	174.20 177.93 229.76 90 90 90	174.34 176.73 225.21 90 90 90
Total reflections	1055651 (47886)		425739 (42117)
Unique reflections	153386 (15196)	306753 (30690)	212977 (21059)
Multiplicity	6.9 (6.4)	3.0 (2.9)	2.0 (2.0)
Completeness (%)	99.97 (99.97)	96.59 (94.09)	99.70 (99.91)
Mean I/sigma(I)	11.5 (1.1)	3.0 (1.5)	3.88 (1.17)
Wilson B-factor	16.10	24.57	34.69
R-pim (all I+ & I-)	0.042 (0.685)	0.223 (1.483)	0.1162 (0.6269)
CC1/2	0.999 (0.431)	0.931 (0.150)	0.989 (0.641)
Refinement statistics			
Reflections used in refinement	153367 (15192)	303998 (29370)	212390 (21040)
Reflections used for R-free	7575 (705)	15065 (1432)	10830 (1062)
R-work	0.1566 (0.2736)	0.2068 (0.3059)	0.2348 (0.3156)
R-free	0.1886 (0.2933)	0.2529 (0.3713)	0.2804 (0.3584)
Number of non-hydrogen atoms	8676	50865	48047
macromolecules	7310	47076	46600
ligands	82	756	383
solvent	1284	3033	1064
Protein residues	889	6177	6150
RMS (bonds)	0.019	0.004	0.002
RMS (angles)	1.69	0.72	0.59
Ramachandran favored (%)	98.20	96.77	96.52
Ramachandran allowed (%)	1.69	3.13	3.08
Ramachandran outliers (%)	0.11	0.10	0.39
Rotamer outliers (%)	0.37	0.90	2.82
Clashscore	3.69	3.53	6.22
Average B-factor	22.77	27.02	39.66
macromolecules	20.27	26.39	39.65
ligands	31.19	43.59	50.38
solvent	36.44	32.54	36.02

Statistics for the highest-resolution shell are shown in parentheses.

Supplementary Table 2: Activity of MMV1557817 against *P. falciparum* gametocytes (IC_{50} values (nM); mean ± SD)

Compound	Early-stage Gametocytes	Late-stage Gametocytes	Mature-stage Gametocytes
**MMV1557817**	**99 ± 2.0**	**309 ± 1.0**	**1474 ± 304**
artesunate	16 ± 5.0	26 ± 2.0	51 ± 23
pyrimethamine	N/A at 40μM	N/A at 40μM	N/A at 40μM
chloroquine	157 ± 3.5	99% at 40μM	94% at 40μM
pyronaridine	67 ± 7.0	2280 ± 679	1254 ± 120
puromycin	163 ± 18	94 ± 5.0	73 ± 24
DHA	16 ± 2.0	35 ± 12	103 ± 30
Methylene Blue	110 ± 4.0	52 ± 2.0	63 ± 26

Supplementary Table 3: Interaction between artemisinin and MMV1557817 against *P. falciparum* Dd2 parasites

Ratio (MMV1557817: artemisinin)	MMV1557817 mean FIC ± SEM	artemisinin mean FIC ± SEM	Σ FICs (interaction)
**0:5**	0	1.00 ± 0.12	
**1:4**	0.87 ± 0.08	0.75 ± 0.07	1.62 (Additive)
**2:3**	1.13 ± 0.19	0.37 ± 0.06	1.49 (Additive)
**3:2**	1.10 ± 0.20	0.16 ± 0.03	1.26 (Additive)
**4:1**	1.05 ± 0.18	0.06 ± 0.01	1.11 (Additive)
**5:0**	1.00 ± 0.19	0	

FIC: fractional inhibitory concentration

Supplementary Table 4: Baseline characteristics of isolates for which *ex vivo* assay was accomplished.

Baseline characteristics	***P. falciparum*** **n=15**	***P. vivax*** **n=8**
**Total number of isolates reaching harvest (%)**	14 (93)	6 (75)
**Median (range) duration of assay (hours)**	44 (41-46)	47 (44-48)
**Geometric mean (95% CI[a]), parasitaemia (asexual parasites/µL)**	31,779 (14,914-67,713)	9,837 (3,124 – 30,975)
**Median initial % (range) of parasites at ring stage**	100[b]	90 (76-99)
**Mean (95% CI) schizont count at harvest**	50(34-86)	45 (28-55)

[a] CI, confidence interval
[b] No range given (all values were 100%)

Supplementary Table 5. ***Ex vivo*** **drug susceptibility to MMV1557818 and standard anti-malarials according to species tested.**

**Drug**	***P. falciparum*** **median EC_{50} (range), nM (n = 15)**	***P. vivax*** **median EC_{50} (range), nM (n = 6)**
**MMV1557817**	98.1 (30.4 – 201.1)	68.6 (57.2 – 150.9)
chloroquine	64.8 (38.3 – 283.2)	36.4 (11.6 – 114.0)
piperaquine	60.8 (17.6 – 130.0)	46.6 (15.0 – 134.8)
mefloquine	10.0 (4.9 – 41.9)	11.2 (8.1 – 20.7)
artesunate	1.2 (0.4 – 4.3)	0.6 (0.3 – 2.4)

Supplementary Table 6: Inhibition of aminopeptidase activity with increasing concentrations of MMV1557817 or artesunate.

**[Compound] (µM)**	***Pf*-M1**	***Pv*-M1**	***Pb*-M1**	**LTA4H**	**ERAP1**	**ERAP2**
**MMV1557817**						
1.25	83 %	96 %	94 %	18 %	0 %	7 %
10	100 %	100 %	100 %	16 %	0 %	0 %
500	-	-	-	71 %	75 %	99.1 %
1000	-	-	-	87 %	98 %	100 %
**Artesunate**						
1.25	4.8 %	2.1 %	0 %	0 %	0 %	0 %
10	11.9 %	23.6 %	12.8 %	12.8 %	0 %	0 %
500	16.8 %	74.0 %	81.3 %	20.2 %	0 %	0 %
1000	43.2 %	68.2 %	78.2 %	36 %	0 %	0 %

Supplementary Table 7: Percent inhibition of control enzyme activity.

**Protein**	**Mean % inhibition by MMV1557817 (n=2)**
HDAC1	2.8
HDAC2	3.0
HDAC5	-2.8
HDAC7	7.7
HDAC8	6.3
HDAC9	-3.6
HDAC10	5.3
MMP-2	39.8
MMP-3	-0.8
MMP-7	-6.7
**MMP-8**	**52.6**
MMP-9	46.3
MMP-14	24.1

Supplementary Table 8: Physicochemical and *in vitro* stability properties of MMV1557817

**Parameter**	**MMV1557817**
MW	394.39
calc Log $D_{7.4}$	3.6
Kinetic Solubility (µg/mL) pH 2.0 / 6.5	12.5-25 / 12.5-25
in vitro microsomal CL_{int} (µL/min/mg) H / R / M*	11 / 29 / 23
in vitro hepatocyte CL_{int} (µL/min/10^6 cells) H / R*	2 / 4
Plasma stability (% remaining, 4 h, 37°C) H / M*	97 / 93
Plasma protein binding (%) H / R / M*	98.8 / 97.8 / 97.7
Albumax media binding (%)	84.5

*H: human, R: rat, M: mouse

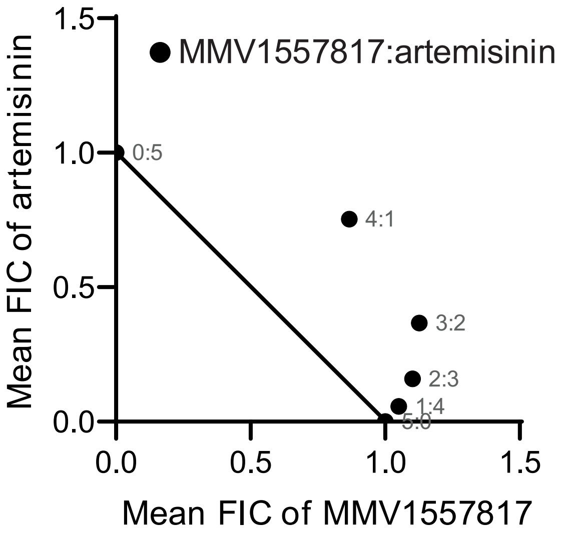

Supplementary Figure 1. Isobologram showing the interaction between **MMV1557817** and artemisinin tested in Dd2 parasites. Fractional inhibitory concentration (FIC) ≤2≥1 indicate additive effects of the individual compounds. n = 3 performed in triplicate.

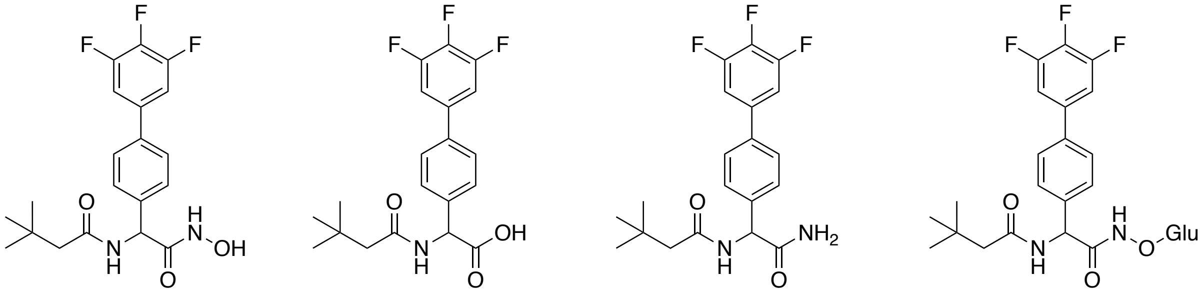

MMV1557817 M-15 M-16 M+176

Supplementary Figure 2. **MMV1557817** and putative metabolites detected following incubation with human and rat cryopreserved hepatocytes.

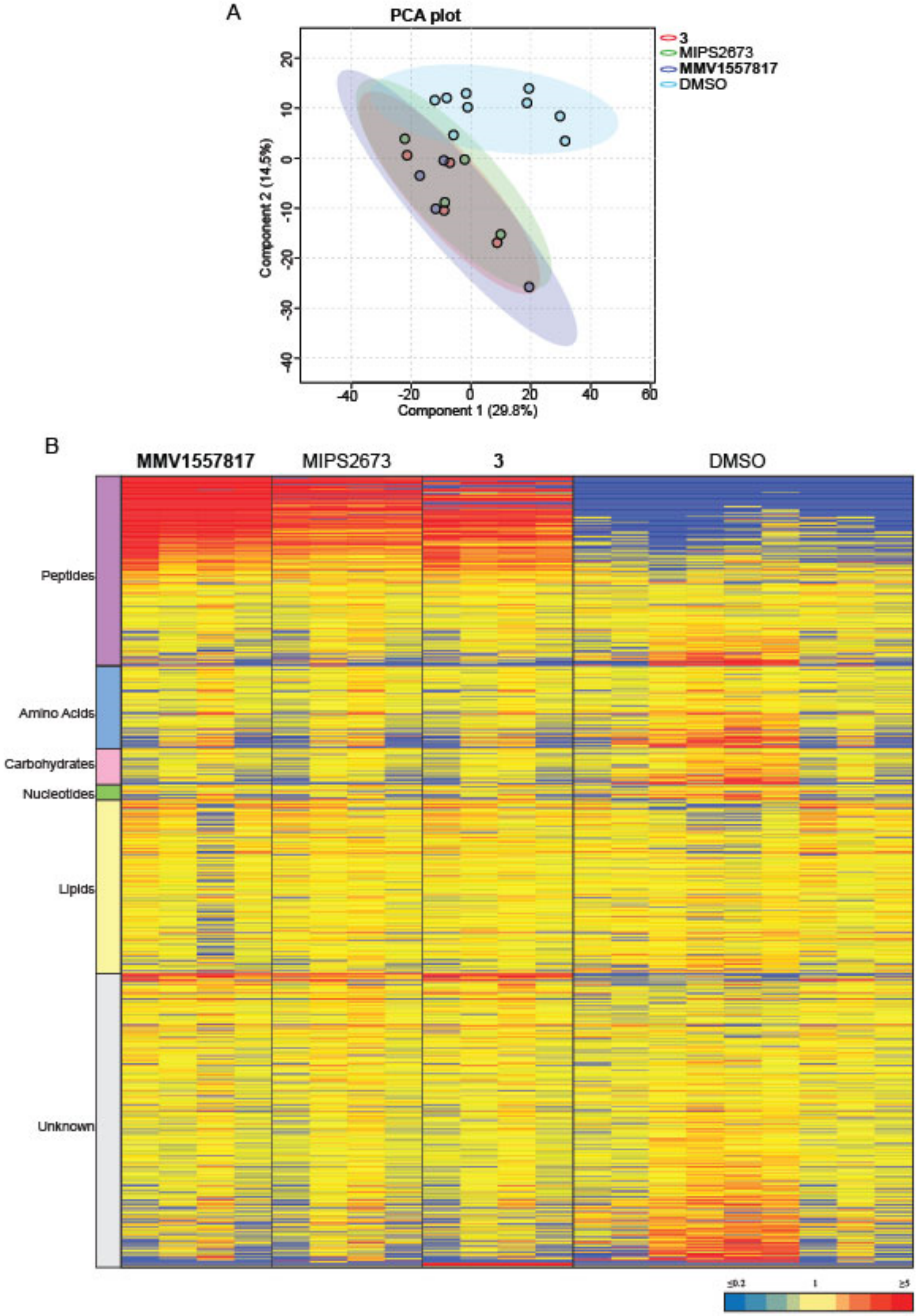

Supplementary Figure 3. Untargeted metabolomics analysis of *Pf*3D7 parasites treated with **MMV1557817**, MIPS2673 (*Pf*A-M1 inhibitor), **3** (*Pf*A-M17 inhibitor), and DMSO control. (A) Principal component analysis (PCA) of parasites treated with

MIPS1557817, MIPS2673 (*Pf*A-M1 inhibitor (53)), **3** (*Pf*A-M17 inhibitor (7)), and DMSO control. Scores plot shows principal components one and two, data points indicate individual sample replicates within each condition and the shaded area denotes 95% confidence interval. (B) Heatmap analysis of peak intensities of all putative metabolites for each condition. Data is shown from 4-7 biological replicates, red, blue and yellow indicates increase, decrease and no change in the relative abundance of putative metabolites identified, respectively. Data for **3** and MIPS2673 has been previously published (7, 53).

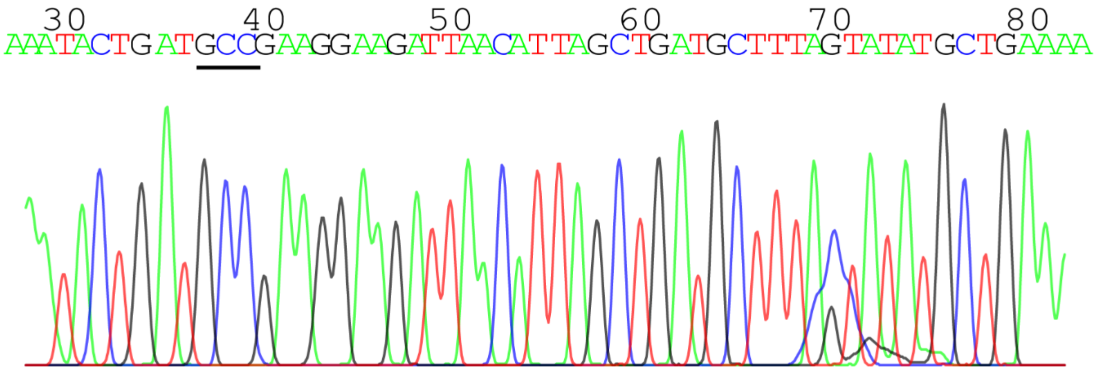

Supplementary Figure 4. Chromatogram of Sanger sequencing of DNA extracted from parasites harbouring a silent mutation (gct to gcc; underlined in black) at A460 in *Pfa-m17* introduced using CRISPR-Cas9.

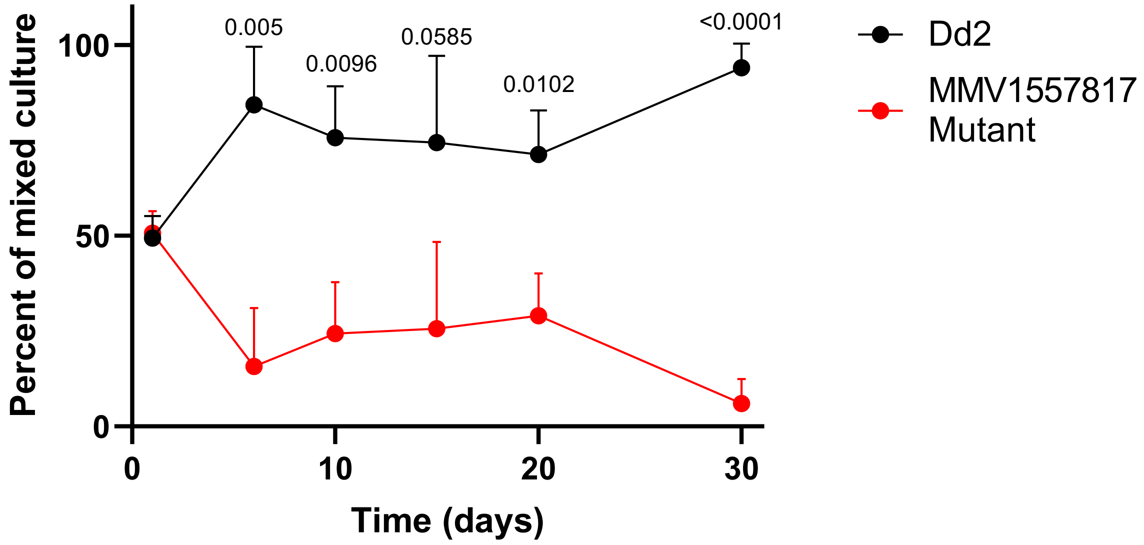

Supplementary Figure 5. Growth competition analysis of **MMV1557817** resistance parasites versus Dd2 as identified through SNP identification by Sanger Sequencing of the A460 locus of *Pfa-m17*. Statistical significance indicated above and determined for each timepoint using an unpaired t-test, n = 3.

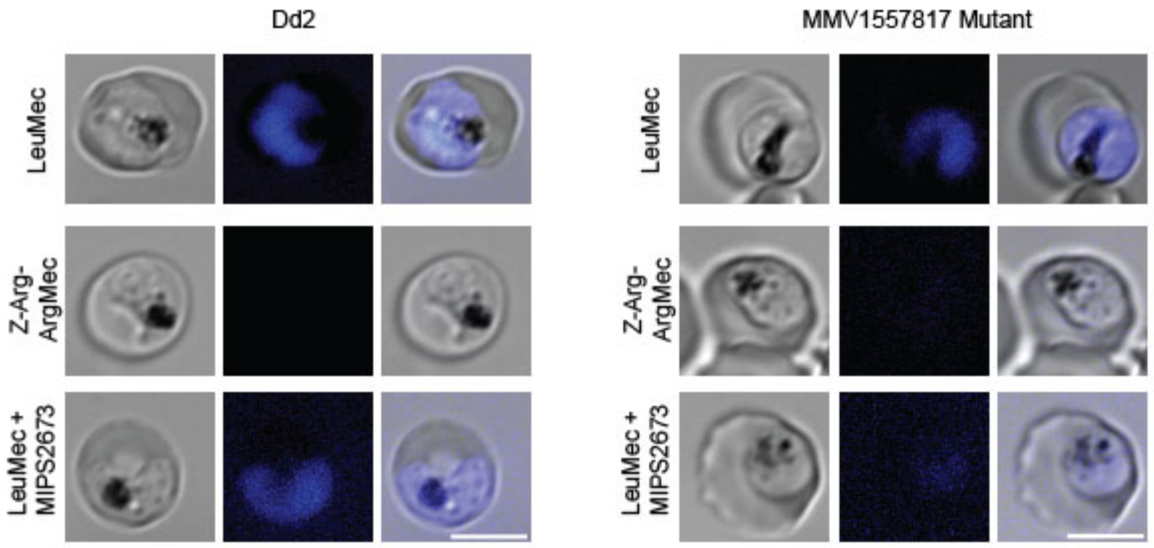

Supplementary Figure 6. Representative images of live cell Dd2 and **MMV1557817** resistant parasites after treatment with the indicated fluorescent substrates. The bottom panel has additionally been treated with the *Pf*A-M1 specific inhibitor MIPS2673 at 10x EC_{50} (3.2 μM; (53)). Scale bar 5 μM.
